# Plasmalogens Regulate Retinal Connexin 43 Expression and Müller Glial Cells Gap Junction Intercellular Communication and Migration

**DOI:** 10.3389/fcell.2022.864599

**Published:** 2022-03-31

**Authors:** Rémi Karadayi, Julie Mazzocco, Laurent Leclere, Bénédicte Buteau, Stéphane Gregoire, Christine Belloir, Mounzer Koudsi, Pauline Bessard, Jean-Baptiste Bizeau, Elisabeth Dubus, Claire Fenech, Loïc Briand, Lionel Bretillon, Alain M. Bron, Xavier Fioramonti, Niyazi Acar

**Affiliations:** ^1^ Eye and Nutrition Research Group, CSGA, Université de Bourgogne Franche-Comté, Dijon, France; ^2^ Taste and Olfaction Research Group, CSGA, Université de Bourgogne Franche-Comté, Dijon, France; ^3^ Brain Nutrient Sensing and Energy Homeostasis, CSGA, Université de Bourgogne Franche-Comté, Dijon, France; ^4^ Department of Ophthalmology, University Hospital, Dijon, France; ^5^ INRAE, UMR NutriNeuro, Bordeaux, France

**Keywords:** plasmalogens, retina, müller cells, connexin 43, gap junctional intercellular communication, cell migration

## Abstract

Plasmalogens are a specific glycerophospholipid subtype characterized by a vinyl-ether bound at their *sn-*1 moiety. Their biosynthesis is initiated in the peroxisome by dihydroxyacetone phosphate-acyltransferase (DHAPAT), which is encoded by the *DAPAT* gene. Previous studies have shown that plasmalogen-deficient mice exhibit major physiological dysfunctions including several eye defects, among which abnormal vascular development of the retina and a reactive activation of macroglial Müller cells. Interestingly, plasmalogen deficiency in mice is also associated with a reduced expression of brain connexin 43 (Cx43). Cx43 is the main connexin subtype of retinal glial cells and is involved in several cellular mechanisms such as calcium-based gap junction intercellular communication (GJIC) or cell migration. Thus, the aim of our work was 1) to confirm the alteration of Cx43 expression in the retina of plasmalogen-deficient DAPAT^−/-^ mice and 2) to investigate whether plasmalogens are involved in crucial functions of Müller cells such as GJIC and cell migration. First, we found that plasmalogen deficiency was associated with a significant reduction of Cx43 expression in the retina of DAPAT^−/-^ mice *in vivo*. Secondly, using a siRNA targeting DHAPAT *in vitro*, we found that a 50%-reduction of Müller cells content in plasmalogens was sufficient to significantly downregulate Cx43 expression, while increasing its phosphorylation. Furthermore, plasmalogen-depleted Müller cells exhibited several alterations in ATP-induced GJIC, such as calcium waves of higher amplitude that propagated slower to neighboring cells, including astrocytes. Finally, *in vitro* plasmalogen depletion was also associated with a significant downregulation of Müller cells migration. Taken together, these data confirm that plasmalogens are critical for the regulation of Cx43 expression and for characteristics of retinal Müller glial cells such as GJIC and cell migration.

## 1 Introduction

The ether-lipid plasmalogens represent a specific glycerophospholipid subgroup that is characterized by the presence of a vinyl-ether bond at the *sn-*1 position of the glycerol backbone. They are synthesized by a multi-step process that starts in the peroxisome with the first and key enzyme of plasmalogen biosynthesis DHAPAT (dihydroxyacetone phosphate-acyltransferase) (Liu et al., 2005). Plasmalogens are present in various concentrations in all cell types and tissues. The heart and nervous tissues are particularly enriched in plasmalogens, where they can make up to 20–30% of total phospholipids (Nagan and Zoeller, 2001). As an extension of the central nervous system, the retina also contains high amounts of plasmalogens, which are mostly represented in the ethanolamine phospholipids subclass (PE) and can make up to 30% of total PE species (Dorman et al., 1976; Acar et al., 2007; Nagy et al., 2012). Interestingly, plasmalogen-deficient mice exhibit several eye defects, including retinal vascular development abnormalities (Saab et al., 2014), as well as a downregulation of brain connexin 43 (Cx43) (Rodemer et al., 2003).

Cx43 is considered as being ubiquitously distributed and is the major connexin in mammal cells (Laird, 2006). Cx43 is implicated in several key cellular functions of glial cells such as gap junction-intercellular communication (GJIC) (Goodenough et al., 1996) or cell migration (Homkajorn et al., 2010). In the retina, Müller cells represent the main glial cell type as they can make up to 90% of macroglial cells (Bringmann et al., 2006). Müller cells are involved in crucial features of the retina, including water and ions homeostasis, angiogenesis, and inflammation (Bringmann et al., 2006; Reichenbach and Bringmann, 2013; Vecino et al., 2016; Toft-Kehler et al., 2018). Such abilities require multiple means of communication between Müller cells, but also with other cell types. For instance, it has been shown that Müller cells can communicate directly through gap junctions-dependent pathways, namely GJIC (Newman and Zahs, 1997; Newman, 2001). Gap junctions are intercellular channels that allow for calcium waves to propagate between adjacent cells among cell networks (Herve and Derangeon, 2013). In the retina, gap junctions between glial cells mostly rely on Cx43 (Zahs and Ceelen, 2006; Kerr et al., 2010), including with astrocytes, which are critical for proper vascular development of the retina (O'Sullivan et al., 2017).

Interestingly, previous data from our laboratory revealed high plasmalogen levels in the retina and suggested that Müller cells play a leading role in plasmalogens biosynthesis (Acar et al., 2007). Thus, using complementary *in vivo* and *in vitro* approaches targeting DHAPAT, our study aimed at determining the impact of plasmalogen depletion on Cx43 expression in the retina as well as on key functional tasks of Müller cells.

## 2 Materials and Methods

### 2.1 Animals and Cells

#### 2.1.1 Animals

Experiments on animals were performed in accordance with the ARRIVE guidelines, the Association for Research in Vision Ophthalmology (ARVO) statement for the use of animals in ophthalmic and vision research, and with French legislation (personal authorization number 21CAA086 for N.A and animal quarters agreement number A21231010 EA), after approval by ethics committees (#105 Comité d’Ethique de l’Expérimentation Animale Grand Campus Dijon) and by the French Ministry of Higher Education and Research (reference number 02271.1).

#### 2.1.2 Müller Cells

**FIGURE 1:**
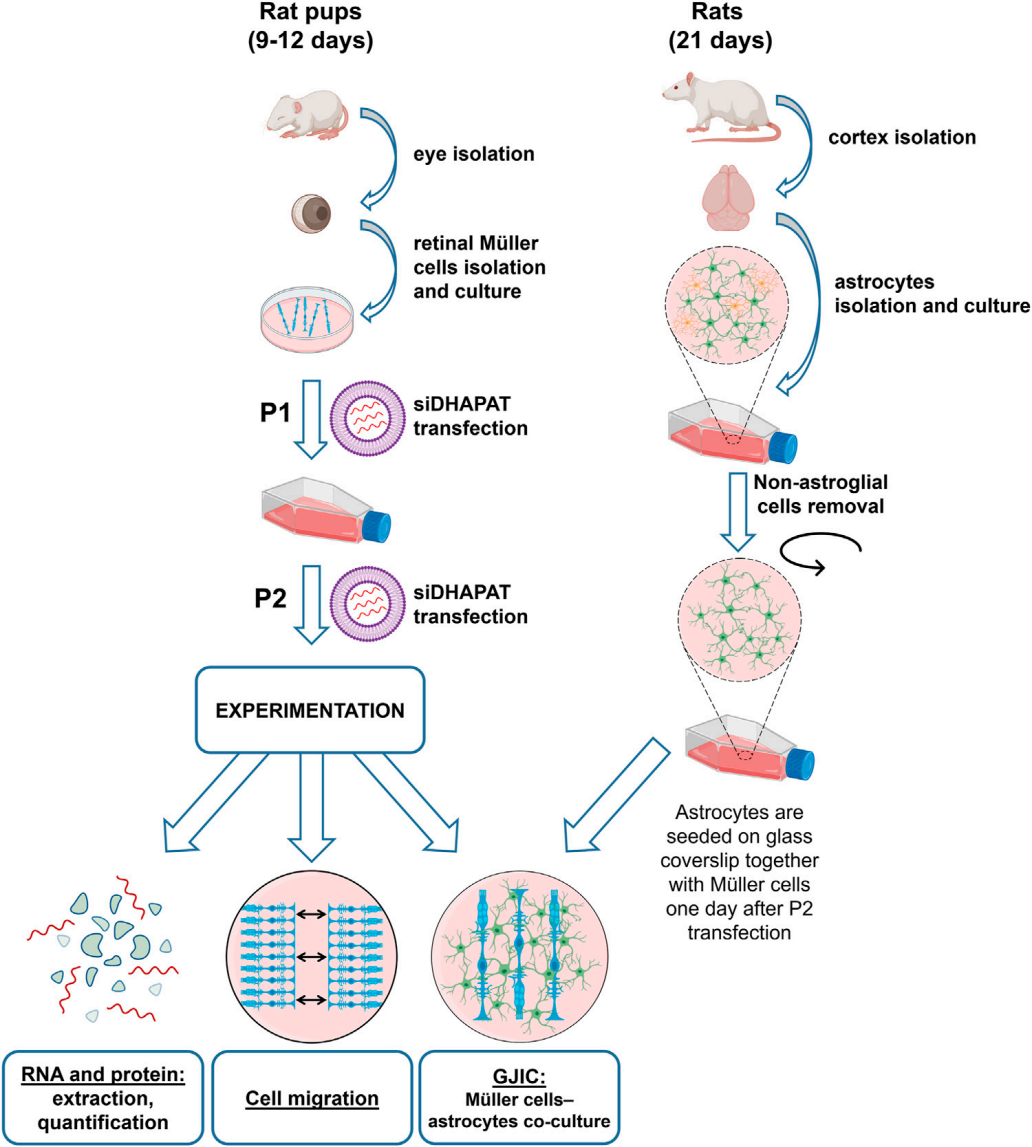
Experimental procedure of plasmalogen depletion using a siRNA-based approach targeting DHAPAT in primary rat retinal Müller cells. Primary Müller cells were prepared from rat retinas according to a previously described procedure (Hicks and Courtois, 1990) with slight modifications. Briefly, eyes were isolated from 9 to 12 days-old rats. Isolated retinas were then dissociated into small pieces and placed into a petri dish. Cells at 80% confluence were reseeded (P1) in a 75 cm^2^ culture flask and transfected with a control or DHAPAT-targeting siRNA depending on their final use. The procedure was repeated until the cells have received a second transfection and were then used at second generation (P2). See Materials and methods 2.1 for the complete primary Müller cells and astrocytes isolation, transfection and co-culture procedure.

Primary Müller cells were prepared from rat retinas according to a procedure previously described by Hicks and Courtois with slight modifications (Hicks and Courtois, 1990). Nine to 12 days-old animals were obtained from a Wistar rat colony established in our animal quarters. Animals were killed by decapitation. Eyes were isolated and placed in Dulbecco’s Modified Eagles Medium (DMEM; Pan Biotech, Germany) with 10% Fetal Bovine Serum (FBS) overnight at room temperature in the dark. Eyeballs were then pre-digested in DMEM containing 0.1% Trypsin-EDTA 10X (Sigma-Aldrich, United States) and 70 U/ml collagenase (Sigma-Aldrich, United States) at 37°C for 45 min. Eyeballs were then placed in DMEM—10% FBS, corneas were incised, lenses and vitreous bodies taken out, and retinas were gently isolated. Retinas were dissociated into small pieces and placed into a Petri dish containing DMEM with 4.5 g/L of glucose, 10% FBS and 10 μL/ml of streptomycin (10 mg/ml)—penicillin (10,000 U/mL) (Pan Biotech, Germany). The medium was not changed during the first 2 days and then progressively substituted by DMEM with 1 g/L of glucose. Cells at 80% confluence were reseeded on different materials depending on their final use (P1) (Figure 1). They were used at second generation (P2).

#### 2.1.3 Astrocytes

Primary astrocytes were extracted from rat cerebral cortex. Twenty-one days-old pups were obtained from the same Wistar rat colony established in our animal quarters. Anesthesia was performed *via* an intraperitoneal injection of pentobarbital (Ceva, France) at 0.1 mg/100 g. Animals were perfused through heart’s left ventricle with a cold PBS solution. The brain was removed and astroglial cells isolation was performed according to a previously described protocol (Schildge et al., 2013) with slight modifications. Briefly, cortices were separated from the brain and the meninges removed. Cortices were cut into small pieces using sharp blades, and cells were then dissociated by three successive trituration and sedimentation steps in DMEM F12 + 10 μL/ml of streptomycin-penicillin medium. Dissociated cells were then plated onto a 75 cm^2^ culture flask, previously pre-coated with poly-L-lysine (1X) and maintained in culture medium (DMEM F12 + 10% FBS +10 μL/ml of streptomycin-penicillin) in an incubator at 37°C with 5% CO2 and 95% humidity. The media was changed every 3–4 days. At 80% confluence, the flask was shaken overnight at 200 rpm to remove non-astroglial cells.

#### 2.1.4 Co-Culture Model

At second generation, Müller cells and astrocytes were seeded on poly-L-lysine coated glass coverslips (18 mm diameter) in 6-wells plates (25 mm diameter) at 5.10^5^ cells/ml and 8.10^5^ cells/ml, respectively. Müller cells were seeded 1 day before astrocytes to allow the transfection with siRNA. Cells were co-cultured together during 4 days before the experiment in DMEM F12 + 10% FBS +10 μL/ml of streptomycin-penicillin medium (Figure 1).

### 2.2 Immunohistochemistry

Adult C57BL/6 mice (3 months of age, Centre d’Elevage Janvier, France) were euthanized by CO2 exposure. The eyeballs were isolated and fixed overnight in formaldehyde solution (PFA) 4% (Sigma-Aldrich, United States). Lens and cornea were then taken out and eyecups were dehydrated in sucrose baths with gradually increasing sucrose concentrations before being embedded in Tissue-Tek O.C.T. (Sakura Finetek, Netherlands) and frozen into liquid nitrogen. 10 µm-thick cryo-sections were performed on a Leica microtome (CM 3050 S, Leica Microsystemes, France) and were mounted on SuperFrost PlusTM slides (Thermo Scientific, United States). Primary Müller cells were isolated as previously described and seeded on microscope slides fixed for 2 h in PFA 4% and kept in 1X PBS +0.01% thimerosal (Sigma-Aldrich, United States) until further experiments. Prior to antibody labeling, both eye cryo-sections and Müller cells-slides were blocked for 1 h in 1% BSA (Sigma-Aldrich, United States) + 0,1% Triton X-100 (Sigma-Aldrich, United States) + 0,05% Tween (Sigma-Aldrich, United States). They were then incubated overnight at +4°C in the same BSA/Triton/Tween solution with the following primary antibodies: GNPAT at a dilution of 1:250 (PA5-36447, rabbit polyclonal, Invitrogen, United States), and Glutamin synthetase at a dilution of 1:10,000 (MA5-27749, mouse monoclonal, Invitrogen, United States). After 5 consecutive washes in 1X-PBS, samples were then incubated for 1 h at room temperature in BSA/Triton/Tween solution with DAPI (1:200, Sigma-Aldrich, United States), Alexa Fluor 488 (A11001, goat anti-mouse, Invitrogen, United States) and Alexa Fluor 594 conjugated secondary antibodies (A-11032, goat anti-rabbit, Invitrogen, United States). Samples were then washed again with 5 consecutive 5 min PBS before being sealed in mounting media (Dako, Denmark) for imaging on an inverted confocal microscope (TCS SP8, Leica Microsystemes, France).

### 2.3 Inhibition of DHAPAT-Mediated Plasmalogen Biosynthesis by siRNA Transfection

Silencer^®^ Select siRNAs were used to knockdown expression of DHAPAT. DHAPAPT-targeting siRNA (siDHAPAT) (siRNA ID s136618 sense 5′-CAU​CGU​UCU​CAA​UUC​UGA​Att-3′, anti-sense 5′-UUC​AGA​AUU​GAG​AAC​GAU​Gga-3′ and siRNA ID s136619 sense 5′-GGA​UGU​CCU​UCA​GUU​GCU​UUt​t-3′, anti-sense 5′-AAG​CAA​CUG​AAG​ACA​UCC​tc-3′) and the non-targeting controls (siCTL) were purchased from Ambion (references 4390771 and 4404020, ThermoFisher Scientific, United States). The siRNA transfection of Müller cells was performed using Lipofectamine RNAiMAX transfection reagent (ThermoFisher Scientific, United States) in OPTIMEM medium (ThermoFisher Scientific, United States) overnight at 37°C in 5% CO2 humidified atmosphere according to manufacturer’s instructions. Medium was then replaced by DMEM supplemented with 10% FBS on the next morning. Finally, cells were used for experiments 5 days after the second transfection.

### 2.4 Evaluation of Müller Cells Plasmalogen Content

Total lipid from Müller cells were extracted according to the method described by Folch and collaborators (Folch et al., 1957) by using a mixture of chloroform/methanol (2:1, v:v). Lipid extracts were stored at −20°C under inert gas until further analyses. Total lipids previously extracted were transmethylated using boron trifluoride (BF3) in methanol according to Morrison and Smith (Morrison and Smith, 1964). Fatty acid methyl esters (FAMEs; formed by the transmethylation of fatty acids at *sn-*1 and *sn-*2 positions of diacylglycerophospholipids and the *sn-*2 of plasmalogens) and dimethylacetals (DMAs; formed by the transmethylation of the aldehyde aliphatic groups on *sn-*1 position of plasmalogens) were subsequently extracted with hexane and analyzed by gas chromatography on a Trace 1310 gas chromatograph (ThermoScientific, Waltham, MA, United States) using a CPSIL-88 column (100 m × 0.25 mm i. d, film thickness 0.20 µm; Varian, France) equipped with a flame ionization detector. Hydrogen was used as the carrier gas (inlet pressure 210 kPa). The oven temperature was held at 60°C for 5 min, increased at 165°C with a 15°C/min rate, held for 1 min and then to 225°C at 2°C/min and finally held at 225°C for 17 min. FAMEs and DMAs were identified by comparison with their commercial and synthetic standards. The data were processed using the EZChrom Elite software (Agilent Technologies, France) and reported as a percentage of total FAMEs and DMAs. Plasmalogen levels were calculated as 2 x (% of total DMAs) as previously described by Acar and collaborators (Acar et al., 2007).

### 2.5 Protein Expression

Proteins were first extracted from Müller cells in RIPA lysis buffer (ThermoFisher Scientific, United States) with extemporaneous addition of a phosphatase inhibitor (Roche, Sigma-Aldrich, United States) and protease inhibitor (Roche, Sigma-Aldrich, United States) cocktails. After a 30-min centrifugation at 10,000 x g, the supernatant containing proteins was isolated. Protein content was measured by using Pierce BCA Protein Assay Kit (ThermoFisher Scientific, United States) at 562 nm by a multilabel plate reader (Victor 3V, PerkinElmer, United States). Dilutions of known concentrations of BSA were prepared and used to determine standard curve.

Twenty five micrograms of protein extracts were boiled for 5 min in a 4X Laemmli buffer (40% glycerol, 240 mM Tris/HCl pH 6.8, 8% SDS, 0.04% bromophenol blue, 5% beta-mercaptoethanol) and then separated by electrophoresis at 125V for 1 h (Mini-PROTEAN^®^ Tetra System, BioRad, United States) using 4–15% SDS-PAGE Stain-Free precast gels (Mini-PROTEAN^®^, TGX Stain-Free, BioRad, United States). After UV activation of the protein gels on a Chemidoc (BioRad, United States), proteins were subsequently transferred on a nitrocellulose membrane (BioRad, United States) using a Transblot Turbo (BioRad, United States). Membranes were blocked for 1 h at room temperature in a PBS solution with 3% BSA (Sigma-Aldrich, United States) and then incubated for 1 h at room temperature with primary antibodies. Primary antibodies used were 1:1,000 rabbit anti DHAPAT (ref 14931-1-AP, ProteinTech, United Kingdom), mouse anti-Cx43 at 1:1,000 dilution (ref 610062, BD Biosciences, United States), 1:1,000 mouse anti-pCx43 (S368) (ref 52559, Cell Signaling Technology, Netherlands), 1:1,000 rabbit anti-pCx43 (Y265) (ref ab193373, Abcam, United Kingdom), rabbit anti GFAP (1:1,000 dilution, ref sc-6171-R, Santa Cruz Biotechnology, United States), mouse anti β -tubulin (1:2000 dilution, ref 32–26,000, ThermoFisher) and rabbit anti β-actin (1:2000 dilution, ref A2066, Sigma-Aldrich). Membranes were then rinsed for 30 min at room temperature using a mixture of PBS-T (10 mM Na2HPO4, 1,76 mM KH2PO4, 137 mM NaCl, 2,7 mM KCl, and 0.1% Tween 20 w/v) and incubated with HRP-conjugated secondary goat anti-mouse antibodies (reference P0161, Dako, Denmark) for 1 h at room temperature. After another 30min-rinsing, blots were incubated for 2min with ECL reagents (Perkin Elmer, United States) and visualized by chemiluminescence using a charge-coupled device (CCD) camera (Chemidoc, BioRad, United States). Protein expression was then quantified using total protein loads.

### 2.6 Gene Expression

After being grown and transfected as described in 2.1, primary Müller cells were collected from culture flask in PBS solution, snap-frozen in liquid nitrogen and stored at −80°C. Total RNA was isolated from primary Müller cells using the NucleoSpin RNA Plus XS Kit (Macherey-Nagel, France) and quantified using a NanoDrop 2000 (Thermo Scientific, United States). RNA samples were then divided in 10 µL aliquots each containing 100 ng of RNA and stored at −20°C. cDNA was synthesized from RNA samples using the high capacity cDNA reverse transcription Kit (Applied Biosystems, United States) and stored in 20 µL aliquots at −20°C. 2µL of cDNA sample were added to 10 µL of iTaq Universal SYBR^®^ Green Supermix (BioRad, United States) plus the corresponding primers. Finally, qPCR was performed on a StepOnePlusTM (ThermoFisher, United States). The qPCR run consisted of a first step at 95°C for 10 min, followed by 40 cycles of 15 s at 95°C, 1 min at 60°C and 30 s at 72°C. In addition to the Cx43-encoding gene (*Gja1*), *Gusb,* and *B2m* were used as housekeeping genes. Primers sequences were as follows: *Gja1*: Forward 5′-CAG​CTG​TTG​AGT​CAG​CTT​GG - 3′, Reverse 5′-ACA​TGG​GCC​AAG​TAC​AGG​AG-3’; *Gusb*: Forward 5′-CTC​GAA​CAA​TCG​GTT​GCA-3′, Reverse 5′- TCA​TTG​AAG​CTG​CAA​GGG​ACC-3’; *B2m*: Forward 5′-CCG​TGA​TCT​TTC​TGG​TGC​TTG -3′, Reverse 5′-CGG​TGG​ATG​GCG​AGA​GTA​CA-3’.

### 2.7 ATP-Stimulated Müller Cells Calcium Response

Primary Müller cells were isolated, cultured and transfected according to the previously described conditions in 2.1 (Hicks and Courtois, 1990). Cells were seeded in Poly-d-lysine coated 96-wells plastic plates (Corning BiocoatTM, Falcon, United States). Müller cells were loaded for 45 min at 37°C with the calcium probe Fluo-4 AM (Molecular Probes, United States) and the organic anion transporters inhibitor probenecid (Invitrogen, United States) in the same modified HBSS. After incubation, plates were placed in a multi-mode microplate reader FlexStation^®^ 3 (Molecular Devices, United States) together with a 500 µM ATP-containing stimulation plate. Müller cells were then stimulated with ATP using the following injection parameters: volume = 50 μL, speed rate 6 (∼94 μL/s), height = 60 µL. Calcium response was recorded during 90 s with the following recording parameters: sensitivity = 50 readings, PMT high; reading interval = 5.04 sec. Data were recorded and analyzed using the SoftMax Pro 5.4.6 software (Molecular Devices, United States). Three 96-wells plates were used as replicates for each experiment. The mean of the three plates was used for analysis.

### 2.8 Calcium Imaging

Co-cultured astrocytes and Müller cells were loaded for 45 min at 37°C under gentle agitation with 2, 5 µM of Fura-2 acetoxymethylester (Fura-2AM, Molecular Probes, United States) in a modified Hanks buffered saline solution (HBSS: 135 mM NaCl, 5 mM KCl, 1.3 mM CaCl2, 1 mM MgCl2, 10 mM HEPES, 2.5 D-glucose, and pH 7.4) supplemented with 0.002% pluronic acid (P3000MP, Molecular Probes, United States). After incubation, glass plates covered with cells were mounted in a thermostatically regulated microscope chamber on an inverted Olympus microscope (IX 70, Olympus Corporation, Japan) and visualized with a 20X objective. Intercellular calcium waves were induced by the application of 100 µM ATP onto a single Müller cell with an 8.4-µm glass micropipette. Two 100 msec-pulses at 10 psi were delivered with a pneumatic PicoPump (PV830, World Precision Instruments, United States). A real-time movie of intercellular calcium waves of stimulated Müller cell and neighboring astrocytes following stimulation was recorded at 2 Hz for 4 min by alternating excitations at 340 and 380 nm (emission spectrum: 420–600 nm). Images were recorded using a CCD camera with the live acquisition software (TiLL photonics, United States). The 340/380 nm fluorescence ratio was calculated after correction for background fluorescence values. A baseline recording was performed for 2 min before stimulation.

### 2.9 Quantitative Analysis of Intercellular Ca2+ Waves

Each cell was considered as an individual region of interest (ROI). For each ROI, we obtained a curve representing the 340/380 nm fluorescence ratio. Since the fluorescence intensity of Fura-2AM is proportional to calcium concentrations, changes in cytosolic calcium concentrations were inferred from the fluorescence profile of individual cells. The mask of each ROI was imported on ImageJ software (National Institute of Health, United States, https://imagej.nih.gov/ij) to measure the distance between the stimulated Müller cell and the subsequently activated astrocytes. Astrocytes were considered as activated once they reached a 10% increase of the baseline (0.1 x Imax + baseline), and the corresponding latency time was evaluated (activation time). The velocity of calcium wave propagation was calculated and expressed in µm/s. Finally, astrocytes calcium response intensity following their activation was evaluated and expressed as a percentage of increase of the baseline (activation amplitude = ((Imax x 100)/baseline)—100). The change in fluorescence intensity was normalized to the level of baseline fluorescence.

### 2.10 Cell Migration

Müller cells migration was evaluated by a wound healing assay. Müller cells were isolated and generated as previously described (see 2.1 and Figure 1). At P2, cells were seeded in a culture-Insert (Ibidi, Germany) at a density of 1 × 10^4^ cells/well. Inserts were removed 5 days after the second siRNA transfection (P2), thus creating a cell-free gap of 500 µm. Cell migration was monitored under microscope by recording a picture each hour during 72 h. Three fields per insert were observed and the results were averaged. The cell-free area and the percentage of recovery were measured and calculated using the ImageJ software (National Institutes of Health, United States).

### 2.11 Cell Proliferation

Cell proliferation was evaluated by Ki-67 labeling of Müller cells. Briefly, Müller cells were washed with PBS and fixed for 10 min with 4% formaldehyde in PBS. After washes and permeabilization steps with TritonX100 (Sigma-Aldrich), cells were incubated with primary antibodies (mouse anti-ki67, dilution 1:1,000, reference M7248, Dako, Denmark) overnight at 4°C. Then, secondary antibody (reference A11001, Thermo Fischer Scientific, dilution 1:1,000) and DAPI were added and cells were cover slipped using a fluorescence-mounting medium. Fluorescence microphotographs were taken using a Nikon microscope (Eclipse E600, Nikon, France) and a Nikon digital camera (DS-Ri2, Nikon) equipped with the Nikon Nis BR software. Images were analyzed with ImageJ software. Proliferation was evaluated as a ratio between the numbers of cells stained by Ki-67 to total cells stained by DAPI.

### 2.12 Statistical Analysis

Statistical analysis was performed using GraphPad Prism v6.05 (GraphPad software, United States). For gene and protein expression, comparisons between two groups were performed using a non-parametric Mann-Whitney test. For the calcium wave quantitative analysis, Pearson correlation coefficients were determined to assess linear associations between astrocytes activation time and their distance from the ATP-stimulated Müller cell. Radius-dependent comparisons between groups were performed by Mann & Whitney test. A *p* value lower than 0.05 was considered as statistically significant and noted by one star (*). Two (**) and three stars (***) were used for *p* values lower than 0.01 and 0.001, respectively.

## 3 Results

### 3.1 Plasmalogen Deficiency is Associated With Retinal Cx43 Downregulation

**FIGURE 2:**
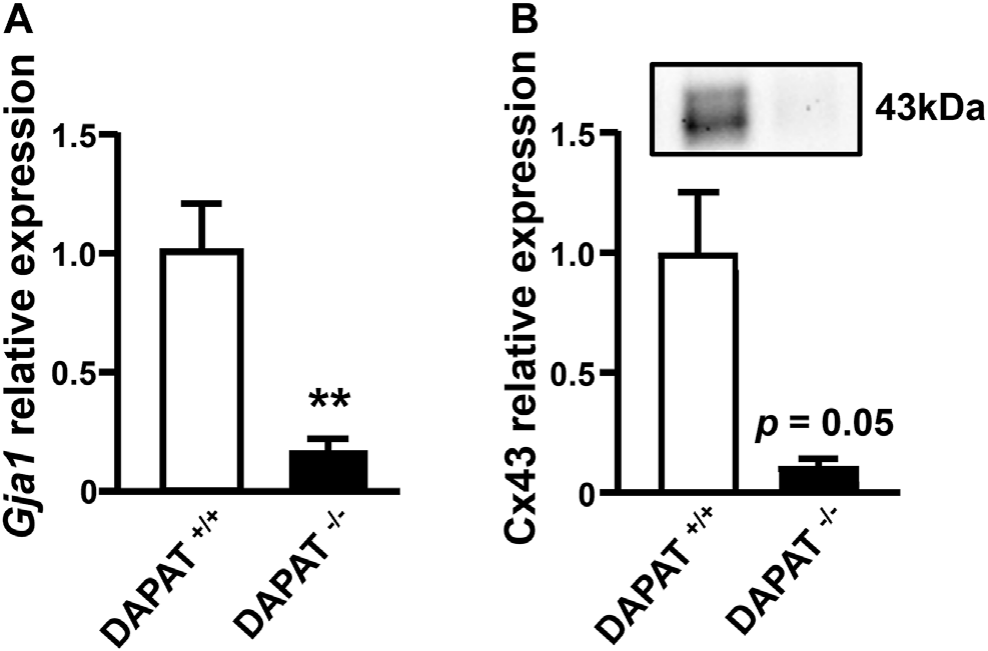
Downregulation of Cx43 gene and protein expression in the retina of plasmalogen-deficient DAPAT adult mice. **(A)** RT-qPCR analysis of *Gja1* expression shows a significant downregulation in 13 weeks-old plasmalogen-deficient DAPAT^−/-^ mice retina compared to DAPAT^+/+^ mice retina (**p < 0.01; n = 6–8). **(B)** Western-blot analysis of Cx43 expression reveals a downregulation in 13 weeks-old plasmalogen-deficient DAPAT^−/-^ mice retina compared to DAPAT^+/+^ retina (*p* = 0.05; *n* = 3). Mann & Whitney test. Data shown as mean ± SEM.

Cx43 gene and protein expression were quantified in 13 weeks-old DAPAT^+/+^ and DAPAT^−/-^ mice retinas. Both Cx43 gene (*Gja1*) and protein were significantly downregulated in a similar fashion, as RT-qPCR revealed a reduction of *Gja1* expression by 83% (*p* < 0.01, *n* = 6–8) (Figure 2A), while western-blots revealed a reduction of Cx43 protein expression by 89% (*p* = 0.05; *n* = 3) (Figure 2B).

### 3.2 Plasmalogens Are Synthesized and Concentrated in Retinal Müller Glial Cells

**FIGURE 3:**
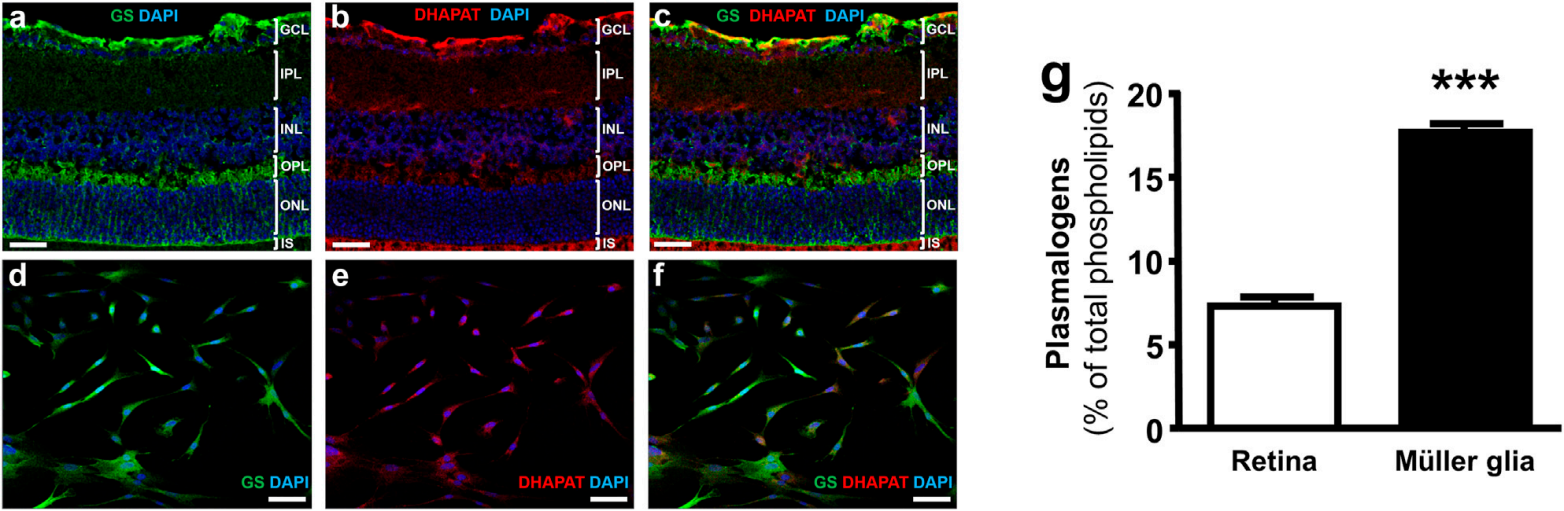
Müller glia express DHAPAT and concentrate plasmalogens in the retina. **(A–C)** Immunohistochemical staining of glutamine synthetase (GS) and DHAPAT in retinal slices. GS expression pattern confirmed the radial Müller cells localization **(A,B)**. DHAPAT-positive cells showed a strong expression pattern in the ganglion cells layer (GCL), the inner nuclear layer (INL) and photoreceptors inner segments (IS) **(B,C)**, corresponding to the localization of Müller cells endfeet, Soma and distal processes, respectively. **(D–F)** Micrographs of primary rat Müller cells labeled with GS and DHAPAT primary antibody showing that GS-positive Müller cells also displayed a strong expression of DHAPAT. **(G)** Plasmalogen quantification by GC-FID reveals a strong concentration in Müller glia compared to whole retina extracts, suggesting a particular function of these ether-lipids in their physiology. *n* = 10. Mann & Whitney test. ***p < 0.001. Data shown as mean ± SEM. GCL = ganglion cell layer; IPL = inner plexiform layer; INL = inner nuclear layer; ONL = outer nuclear layer; OS = outer segments of the photoreceptors. Scale bars = 50 µM.

Immunohistochemical staining of retinal cryosections revealed DHAPAT expression in several layers corresponding to the ganglion cell layer (GCL), the inner nuclear layer (INL) and the photoreceptors inner segments (IS) (Figures 3A,B). Such a pattern is consistent with the localization of Müller cells endfeet, Soma and distal processes, respectively. DHAPAT expression in Müller glia was then further confirmed by immunocytochemistry. A glutamine synthetase (GS)-DHAPAT double immunolabeling first confirmed the purity of our Müller cells culture and that Müller cells strongly express DHAPAT (Figures 3C–F). Finally, in order to assess the extent of plasmalogen biosynthesis activity in Müller glia, plasmalogen concentrations were compared in total lipid extracts from primary rat Müller cells and whole mouse retinas. A significantly higher concentration of plasmalogen in Müller cells compared to whole retina extracts (17.67 ± 0.30% vs. 7.53 ± 0.30% of total phospholipids, *p* < 0.001) (Figure 3G), confirming Müller glia as a major reservoir of plasmalogens in the retina. These data also reinforce the idea that plasmalogens might play specific roles in Müller cells physiology.

### 3.3 DHAPAT-Targeting siRNA Treatment is Associated to a Depletion of Plasmalogen Cell Content

**FIGURE 4:**
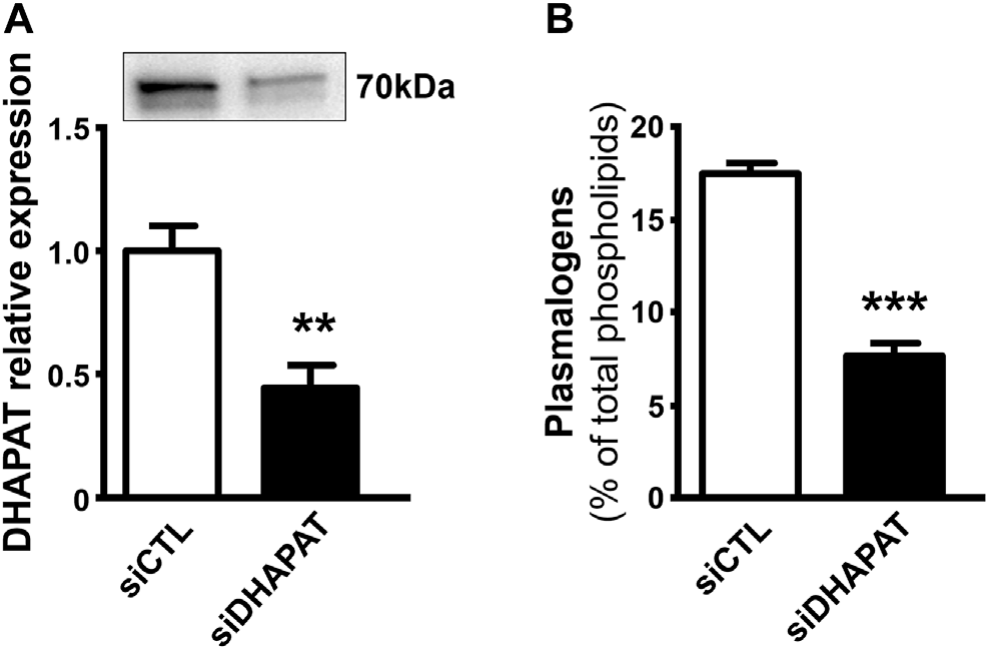
Loss of DHAPAT expression in siDHAPAT-transfected Müller glial cells. **(A)** Western-blot analysis shows a significantly reduced expression of DHAPAT in primary Müller cells transfected with a DHAPAT-targeting siRNA (siDHAPAT) compared to control siRNA-transfected Müller cells (siCTL). **(B)** Plasmalogen concentrations determined by GC-FID are significantly reduced in siDHAPAT-transfected cells. Mann & Whitney test. **p < 0.01; ***p < 0.001. *n* = 10. Data shown as mean ± SEM.

In a second series of experiments, we decided to assess the efficiency of our siRNA-based plasmalogen depletion protocol. Plasmalogen content in Müller cells was assessed by a double transfection of primary Müller cells with a DHAPAT-targeted siRNA (siDHAPAT). siDHAPAT treatment induced a significant decrease in DHAPAT protein expression (−56%, *p* < 0.01) (Figure 4A) as well as a significant 56%-decrease in the cellular plasmalogen content (7.74 ± 0.59% *vs*. 17.61 ± 0.35% of total phospholipids, *p* < 0.001) (Figure 4B).

### 3.4 Plasmalogen Depletion of Müller Cells is Associated With Cx43 Protein Downregulation

**FIGURE 5:**
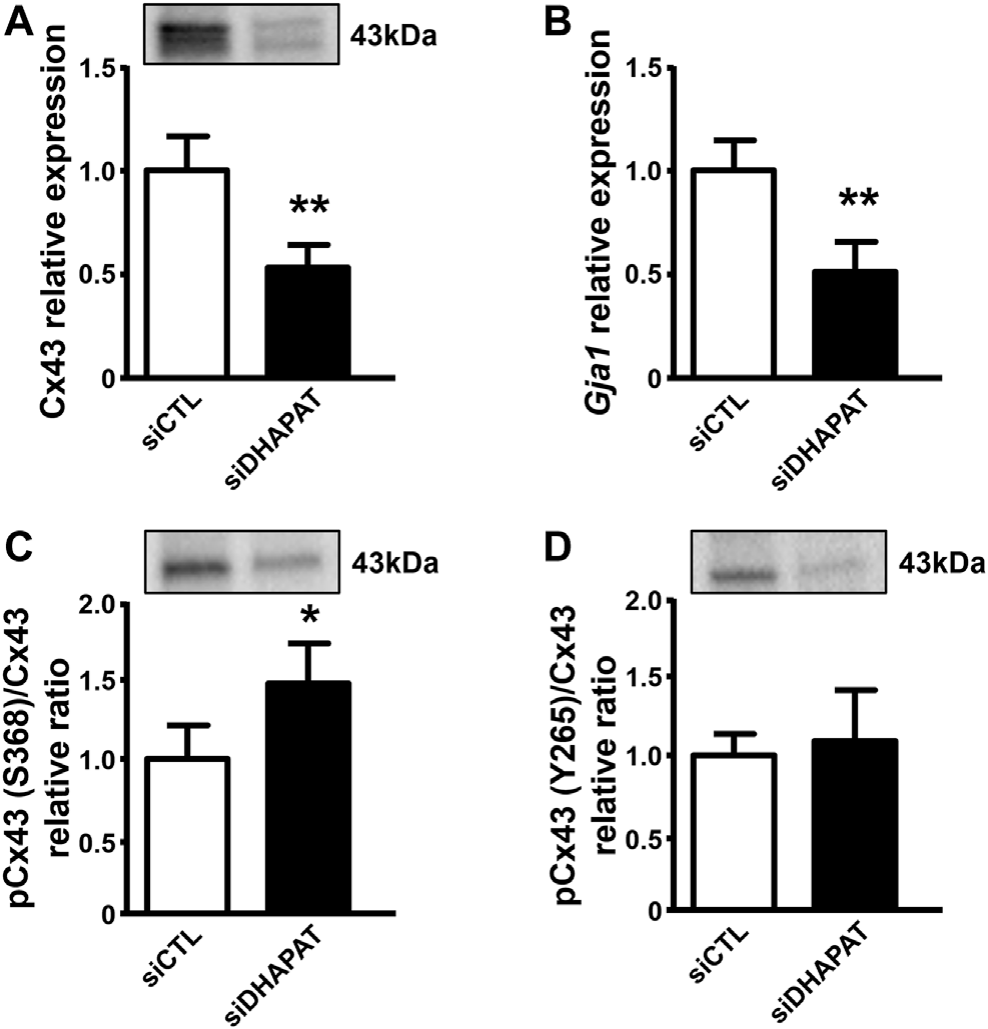
Plasmalogen depletion impacts Cx43 expression in primary Müller cells culture. **(A)** Cx43 protein expression is significantly decreased in siDHAPAT-transfected primary Müller cells. **(B)** Reverse transcription quantitative PCR (RT-qPCR) indicates that siDHAPAT transfection significantly downregulated *Gja1* expression. **(C,D)** Western-blot analyses show a significant increase in the phosphorylation status of Cx43 on its S368 residue **(C)** but not on its Y265 residue **(D)**. *n* = 6–8 cell cultures. Mann & Whitney test. *p < 0.05; **p < 0.01. Data shown as mean ± SEM.

Reduced levels of plasmalogens were significantly associated with a 47%-decrease in Cx43 protein expression (*p* < 0.01) (Figure 5A). RT-qPCR analyses further showed that *Gja1* expression was also significantly downregulated in siDHAPAT-transfected Müller cells (−47%, *p* < 0.01) (Figure 5B). In order to determine whether other Cx43-dependent mechanisms are involved in Cx43 downregulation, we assessed Cx43 phosphorylation status, as it was shown that phosphorylation of Cx43 on S368 and Y265 residues triggers its degradation through the ubiquitin-proteasome pathway (Leithe et al., 2018). We found that plasmalogen depletion was associated with a significant increase by 52% on the S368 (*p* < 0.05) (Figure 5C) but not on the Y265 residue (Figure 5D).

### 3.5 Plasmalogen Depletion Affects Müller Glia Response to ATP

**FIGURE 6:**
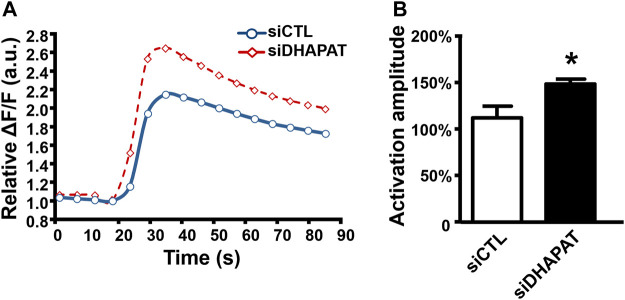
Plasmalogen depletion enhances the calcium response of ATP-stimulated Müller cells. **(A)** Mean kinetic curves responses of control (siCTL) and plasmalogen-depleted (siDHAPAT) primary Müller cells stimulated with ATP. **(B)** Activation amplitude of ATP-stimulated Müller cells show that plasmalogen-depleted Müller cells display a significantly increased calcium response. *n* = 4. Mann & Whitney test. * *p* < 0.05. Data shown as mean ± SEM.

To evaluate whether Cx43 downregulation could be associated with functional defects of Müller cells, we investigated Müller cells direct response to ATP stimulation (Figure 6). Müller cells treated with siDHAPAT exhibited a significant increase of the ATP-mediated calcium response, which was 36%-higher when compared to siCTL-treated cells (149 ± 5% vs. 113 ± 12%, *p* < 0.05) (Figures 6A,B).

### 3.6 Plasmalogen Depletion Alters Retinal Glial Cells Gap Junction-dependent Intercellular Communication

**FIGURE 7:**
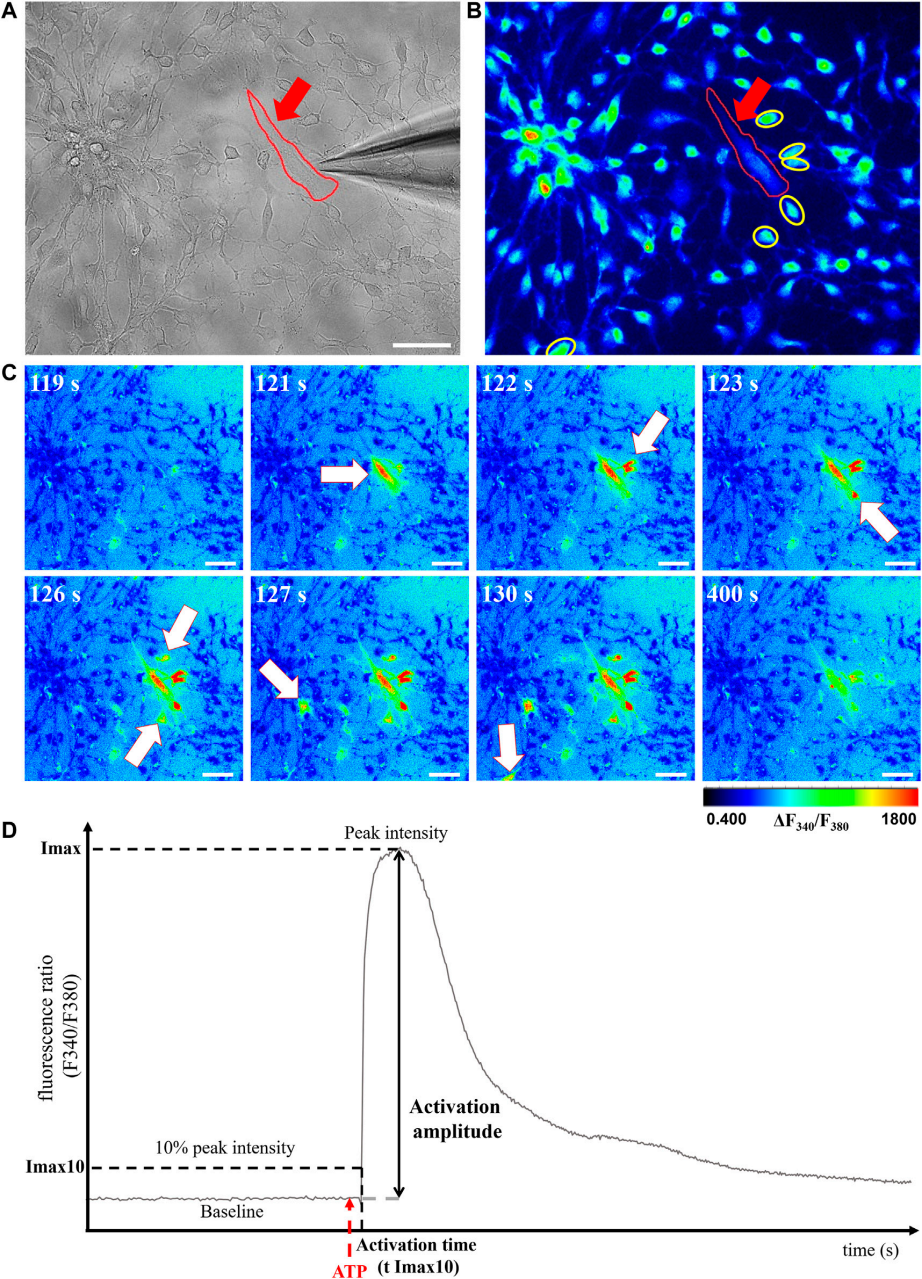
Evaluation of GJIC between retinal Müller cells and astrocytes. **(A,B)** Calcium imaging. Müller cells (red arrow) can be morphologically distinguished from astrocytes (yellow circles) under brightfield **(A)** and fluorescent **(B)** modes. **(C)** ATP-stimulated calcium response of the Müller cell and neighboring astrocytes could be monitored under fluorescent microscopy as a function of time (white arrows). Pseudocolor images were obtained by the 340 nm/380 nm fluorescence ratio and the pseudocolor scale indicates fluorescence ratio values. **(D)** Graphical representation of astrocytes calcium response curves with the parameters selected for further analyses: cell activation threshold (Imax10), activation amplitude (% of baseline increase) and cell activation threshold time (T Imax10) were analyzed. Scale bars = 50 µm.

**FIGURE 8:**
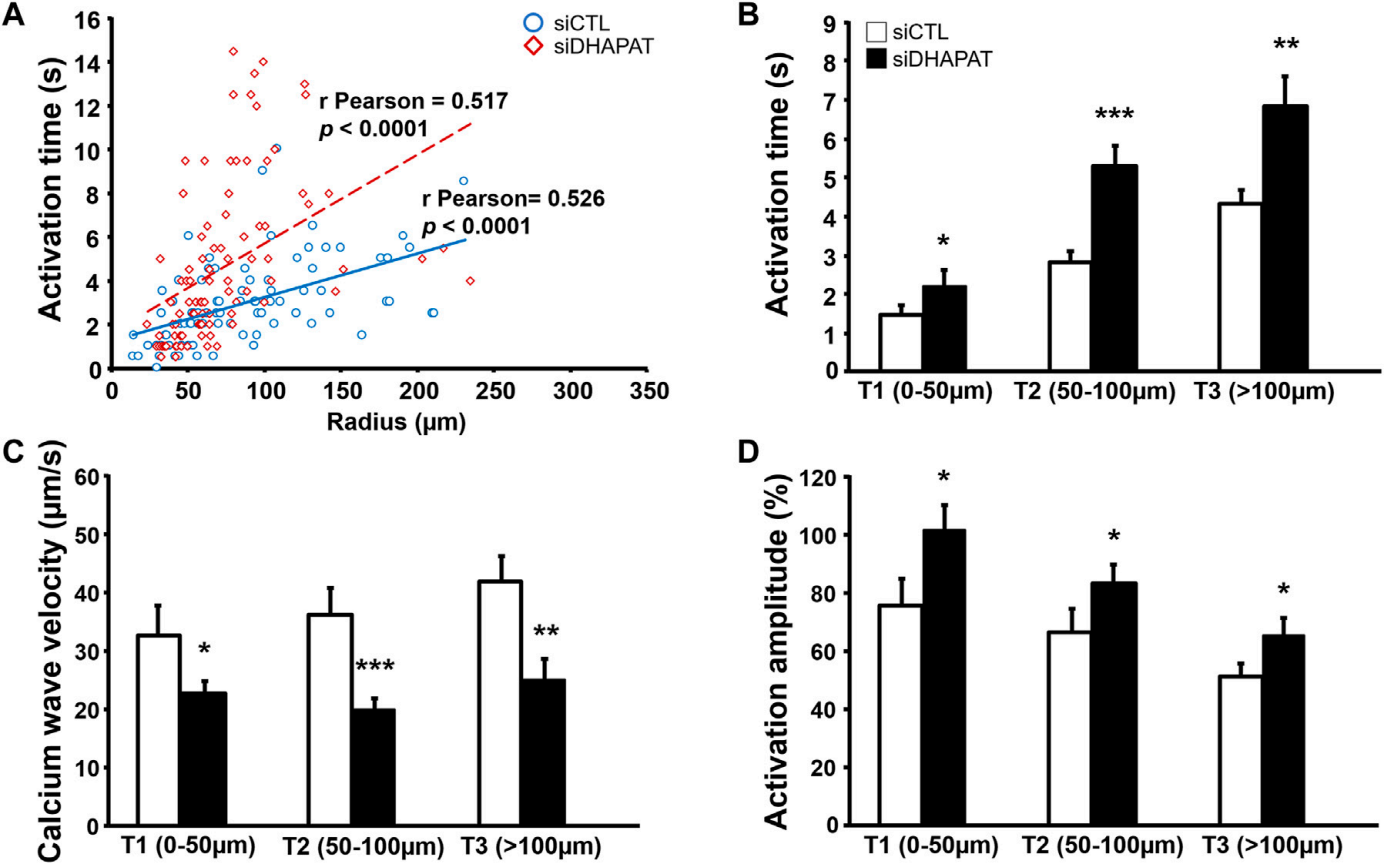
GJIC alterations in astrocytes neighboring plasmalogen-depleted Müller cells. **(A,B)** Astrocytes stimulated by plasmalogen-depleted Müller cells display a significant increase in the activation time **(A,B)** and amplitude **(D)**, as well as in calcium wave velocity in all tertiles of distance **(C)**. n ≥ 75 astrocytes. Student *t* test. *p < 0.05; **p < 0.01; ***p < 0.001. Data shown as mean ± SEM.

In the retina, Müller cells not only engage in GJIC with other Müller cells, but also with astrocytes, which are critical for numerous physiological features, including vascular development (O'Sullivan et al., 2017). We then used calcium imaging to determine whether plasmalogen depletion affects Müller cells-astrocytes intercellular calcium-based communication. siDHAPAT or siCTL-treated Müller cells were selectively activated in response to topical ATP application using a glass micropipette placed adjacently to the cell (Figure 7A). Astrocytes could be morphologically distinguished from Müller cells (Figures 7A,B), and the activation of neighboring astrocytes could be effectively monitored as the calcium wave propagated among the astrocytic network (Figures 7C,D). Astrocytes activation time data were plotted against the radius from the stimulated Müller cell and Pearson’s correlation coefficients were determined to assess the statistical relationship between these two variables. The data showed positive linear associations for each group, confirming that astrocytes activation time is directly correlated to their distance from the ATP-stimulated Müller cell (rPearson: siCTL = 0.517; siDHAPAT = 0.5175) (Figure 8A). To further analyze astrocytes responses, we decided to split these data among 3 radius tertiles according to their distance from the ATP-stimulated Müller cell (T1 = 0–50 µm; T2 = 50–100 µm; T3 > 100 µm). Tertiles analyses confirmed that the increase in activation delay of neighboring astrocytes was maintained as the distance increased from the ATP-stimulated stimulated Müller cell (Figure 8B). Plasmalogen-depletion of Müller cells was associated to an increase of astrocytes activation time (Figure 8B) as well as a decrease of calcium wave velocity (Figure 8C), regardless of the distance between the astrocyte and the Müller cell (0–50 µm, *p* < 0.05; 50–100 µm, *p* < 0.001; >100 µm, *p* < 0.01). ATP stimulation of plasmalogen-depleted Müller cells also led to a significant increase in the activation amplitude of astrocytes whatever their distance to the ATP-stimulated Müller cells (*p* < 0.05) (Figure 8D), suggesting that the previously shown over-activation of plasmalogen-depleted Müller cells (Figure 5) is maintained throughout the calcium propagation to neighboring astrocytes.

Taken together, these results suggest that plasmalogens are involved in the regulation of ATP-stimulated calcium response of retinal Müller cells as well as its propagation through gap junctions to the neighboring astrocyte networks.

### 3.7 Plasmalogen Depletion Alters Müller Cells Migration

**FIGURE 9:**
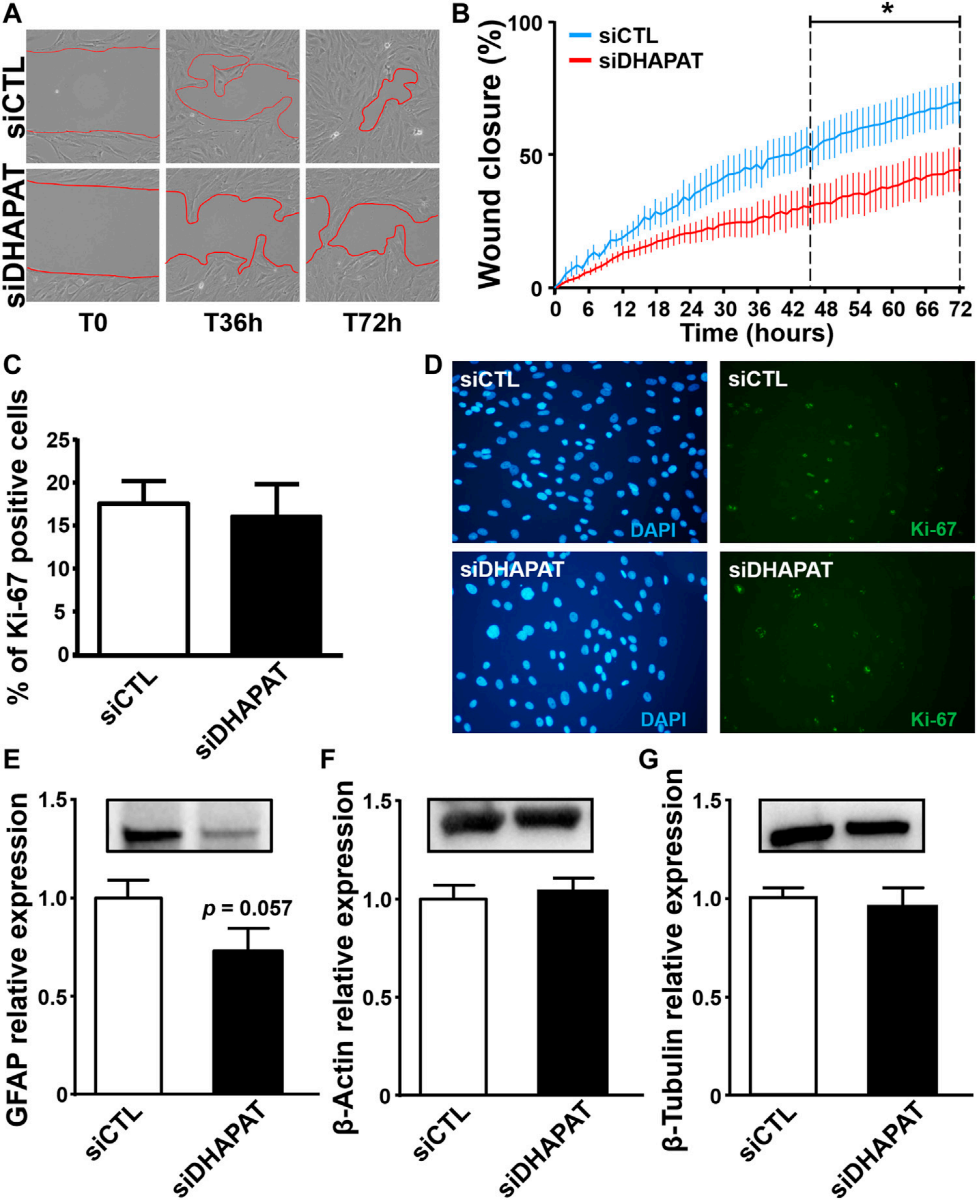
Plasmalogen depletion of primary Müller cells is associated with a decrease of cell migration as well as GFAP expression, but does not modify cell proliferation or other cytoskeleton-related proteins expression. **(A)** Representative micrographs of primary Müller cells treated with a control siRNA (siCTL) or a DHAPAT-targeting siRNA (siDHAPAT) in a wound closure assay before treatment (T0), 36 and 72 h after treatment. **(B)** Quantification of wound closure assays. Summary graph of Müller cells migration speed represented as a percentage of wound closure at indicated time points. Plasmalogen-depleted Müller cells exhibited a significant reduction of migration speed when compared to controls (siDHAPAT vs. siCTL, *p* < 0.05) (*n* = 10 for siCTL, *n* = 9 for siDHAPAT). Two-way ANOVA with Tukey’s multiple comparisons test. **(C)** Müller cells proliferation. Ki-67-labeled Müller cells were quantified as a percentage (%) of DAPI-positive cells. Plasmalogen-depleted Müller cells did not show any alteration of proliferation when compared to controls (siDHAPAT vs. siCTL) (*n* = 10 for siCTL, *n* = 9 for siDHAPAT). **(D)** Fluorescence microphotographs of DAPI and Ki-67-labeled Müller cells in siCTL and siDHAPAT-transfected groups. **(E–G)** Western-blot analyses show a reduction of GFAP expression in siDHAPAT-transfected Müller cells (*p* = 0.05) **(E)**, while other cytoskeleton-related proteins expression such as β-Actin **(F)** or β -Tubulin (g) remained unchanged. *n* = 4-5 cell cultures. Mann & Whitney test. *p < 0.05. Data shown as mean ± SEM.

Through wound healing assays, we observed that the wound closure was about 70% after 72 h for siCTL-treated cells (Figures 9A,B). Inhibition of plasmalogen biosynthesis strongly affected the migration ability of retinal Müller cells as the rate of wound closure was significantly reduced by 35–40% after 72 h in siDHAPAT-treated Müller cells (*p* < 0.05) (Figures 9A,B). Interestingly, plasmalogen depletion was also associated with a 28%-decrease in GFAP protein expression (*p* = 0.057) (Figure 9E), but not that of other cytoskeleton proteins such as β-Actin (Figure 9F) or β-Tubulin (Figure 9G). Finally, alteration of Müller cells migration was not associated with modifications of cell proliferation as plasmalogen-depleted Müller cells did not exhibited any change in Ki-67 labeling (Figures 9C,D).

## 4 Discussion

In this study, we used a double approach that relied on both *in vivo* and *in vitro* models to investigate the role of plasmalogens in Cx43-related functions of Müller cells. First, we used a plasmalogen-deficient mouse model (DAPAT mice) to assess the existence of an association between plasmalogens and Cx43 in the retina, which was previously identified in plasmalogen-deficient mice brains (Rodemer et al., 2003). Our results show that plasmalogen deficiency is associated with a significant downregulation of both Cx43 protein and gene (*Gja1*) in the retina of 13 weeks-old mice. To our knowledge, this is the first time that Cx43 downregulation is highlighted in the retina of adult DAPAT^−/-^ mice, thereby confirming previously published results in the brain and the heart of plasmalogen-deficient mice (Rodemer et al., 2003; Todt et al., 2020) and suggesting that several tissues may face the same dysregulation mechanisms regarding Cx43 expression. Further studies are required to determine if such a Cx43 downregulations takes place progressively during post-natal growth or if mice pups are born with these alterations already in place, which could be another mechanism underlying the vascular development and glial abnormalities described in the developing DAPAT^−/-^ mouse (Saab et al., 2014).

We then confirmed that not only Müller cells do express the plasmalogen-synthetizing enzyme DHAPAT, but that they also concentrate plasmalogens when compared to whole retinal extracts, suggesting a specific role for these ether-lipids in Müller cells. Yet, we did not know to what extent they are significant for their physiological functions. Furthermore, considering that plasmalogen metabolism seem to be associated with Cx43 regulation, we decided to further investigate this association and the main mechanisms involved *in vitro* using primary Müller cells.

Using molecular and biochemical approaches, we showed that plasmalogen depletion was sufficient to trigger Cx43 downregulation by affecting both transcriptional and post-translational mechanisms in Müller cells *in vitro*. *Gja1* expression is known to be regulated by numerous mechanisms such as transcription factors, epigenetic mechanisms (Oyamada et al., 2013), and even vitamin-associated compounds (Vine et al., 2005). It would be of particular interest to conduct dedicated studies in order to determine the mechanisms leading to *Gja1* downregulation in plasmalogen-depleted Müller cells. Moreover, one of the key features of the Cx43 stands to its C-terminus tail. Cx43 C-terminus consists in an unusually long intracytoplasmic tail with many binding sites for several enzymes, making it a target for various regulation mechanisms such as phosphorylation (Leithe et al., 2018), which is known to regulate its degradation by the ubiquitin-proteasome pathway (Leithe and Rivedal, 2004; Kimura and Nishida, 2010). Here we focused on the main phosphorylation sites linked to PKC (S368) and Src (Y265) activities. Our results indicate that plasmalogen depletion resulted in Cx43 overphosphorylation by PKC, but not by Src. Therefore, we suggest that Cx43 degradation is increased by a PKC-mediated overphosphorylation that may be further reducing cell content in Cx43. Cx43 overphosphorylation by PKC in plasmalogen-depleted Müller cells could be a direct consequence of the changes in cell membrane composition. Indeed, previous studies have shown that unlike usual diacyl glycerol (DAG) generated from phospholipids by phospholipase C (PLC) activity, alkenylacyl-glycerides (plasmalogen-derived DAG) does not induce PKC activation despite being ligands of the enzyme (Cabot and Jaken, 1984; Ganong et al., 1986; Heymans et al., 1987; Daniel et al., 1988). Instead, they would rather act as DAG competitive inhibitors, therefore representing an inhibition mechanism for PKC activity (Clark and Murray, 1995; Warne et al., 1995; Mandal et al., 1997). Besides, PKC activation requires prior docking to the cell membrane. Several phospholipid species of the choline (PC) and serine (PS) subgroups, as well as DAG or even phosphoinositides such as phosphatidylinositol bisphosphate (PIP2) are involved in this mechanism (Gould and Newton, 2008; Guerrero-Valero et al., 2009; Lucic et al., 2016). Accordingly, one could hypothesize that plasmalogen depletion induces spatial and molecular rearrangements of the cell membrane, leading to a facilitated recruitment of PKC. Further studies are needed to elucidate the association between plasmalogens and Cx43 expression and phosphorylation in Müller cells.

Moreover, Cx43 being the major component of retinal gap junctions, we aimed to look for potential alterations of GJIC in a two-steps manner: 1) the initiation steps in Müller cells, and 2) propagation of the calcium wave to neighboring cells. By using the Flexstation 3^®^ plate reader device, we were able to precisely study the isolated calcium response of Müller cells to ATP-stimulation with reproducible and well-calibrated parameters. Our data reveal an increase in the activation amplitude of plasmalogen-depleted Müller cells (siDHAPAT = 85% vs. siCTL = 64%). Interestingly, previous research reported a decrease of the activation amplitude in Cx43 KO astrocytes (Scemes et al., 1998). Even though our model only exhibited partial Cx43 depletion, this suggests the existence of some distinct mechanisms between Müller cells and astrocytes to ATP-induced calcium response. To our knowledge, our data are the first reporting an increase of ATP-induced calcium response following a plasmalogen depletion in Müller cells. Interestingly, previous studies have linked calcium release from the endoplasmic reticulum (ER) to n-3 polyunsaturated fatty acids (PUFAs) such as docosahexaenoic acid (DHA) in astrocytes (Sergeeva et al., 2005; Begum et al., 2012) showing that DHA reduces astrocytic calcium response amplitudes, partly by inhibiting the IP3 receptors activity. As plasmalogens are known to be “reservoirs” of PUFAs (including DHA) in the cell membrane (Nagan and Zoeller, 2001), we could hypothesize that plasmalogen depletion is associated to a lower DHA bioavailability in Müller cells, thereby preventing its inhibitory effects on the calcium release from the ER. This hypothesis is supported by a significant reduction of the n-6/n-3 PUFAs ratio in siDHAPAT-treated Müller cells (data not shown), and by a previous work from our laboratory showing that inhibiting PUFAs release from plasmalogens leads to alterations similar to plasmalogen deficiency in mouse (Saab et al., 2014).

Considering that Müller cells also engage in GJIC with astrocytes, which are crucial for vascular development of the retina (Saab et al., 2014) and other key features of retinal physiology like blood flow regulation (Metea and Newman, 2006; Newman, 2015), we aimed at checking for potential alterations of GJIC between plasmalogen-depleted Müller cells and astrocytes. Our data show that siDHAPAT-treated Müller cells evoked calcium waves that propagated slower than calcium waves originating from control Müller cells (Figure 8C), thus resulting in a delayed activation of neighboring astrocytes (Figure 8B). The average activation time of astrocytes closer than 50 µm from the Müller cell was 1.8 s for the siCTL group, while it rose to 2.5 s for the siDHAPAT group. Here, we show that calcium waves from control Müller cells propagated at an average speed of 37.3 μm/s, which is consistent with previous studies (Newman and Zahs, 1997; Scemes et al., 1998) measuring calcium wave velocities of 25.3 μm/s and 18.8 μm/s, respectively. Calcium waves originating from plasmalogen-depleted Müller cells propagated at 23.7 μm/s, which represents a 37%-diminution in propagation velocity. Interestingly, Scemes, Dermietzel and Spray also found a 36% reduction of calcium wave velocity in Cx43-KO astrocytes (Scemes et al., 1998). However, they also found that knocking out Cx43 induced a reduction in astrocytes activation amplitude, while we found that astrocytes stimulated by plasmalogen-depleted Müller cells (with downregulated Cx43) exhibited stronger activation amplitudes (Figure 8D), which seems consistent with the fact that plasmalogen-depleted Müller cells displayed an increase in their ATP-induced calcium response (Figure 6). Accordingly, our results allow us to hypothesize that astrocytes alteration in the plasmalogen-deficient DAPAT^−/-^ mouse could be associated with (at least partly) functional defects of Müller cells. In order to validate our findings, it would be of particular interest to assess Müller cells response to ATP and the subsequent GJIC *in situ* on whole retinas or on isolated Müller cells from *ex vivo* DAPAT^−/-^ mice retinas using previously described procedures (Uckermann et al., 2002; Kurth-Nelson et al., 2009; Tchernookova et al., 2018). Further studies would then be needed to evaluate to what extent the defects in GJIC between Müller cells and astrocytes participate to the abnormal astrocytic template formation in the DAPAT^−/-^ mice as observed previously (Saab et al., 2014).

Besides, the functions of Cx43 in glial cells are not restricted to GJIC since several studies pointed out the implication of Cx43 in other cellular mechanisms such as cell migration (Homkajorn et al., 2010; Lagos-Cabre et al., 2019; Olk et al., 2010). Indeed, Cx43 is a transmembrane protein that acts as a membrane anchor for cytoskeletal proteins (Butkevich et al., 2004; Crespin et al., 2010; Matsuuchi and Naus, 2013), thereby regulating cell cytoskeleton network. Therefore, it seemed likely that alterations of Cx43 may induce modifications of the cytoskeleton network organization and subsequent cell migration (Figures 9A,B), potentially even without altering the expression of its protein actors like β-actin or β-tubulin (Figures 9F,G). Surprisingly, we found that reducing levels of plasmalogens was associated with a downregulation of GFAP (Figure 9E) in Müller cells. As GFAP is overexpressed in activated Müller cells during gliosis and considering its implication in cell migration mechanisms, our data suggest that lowering Müller cells plasmalogen content may influence the reactivity of Müller cells to gliosis but also their ability to migrate. To our knowledge, this is the first time that plasmalogen metabolism is associated with such functional alterations of Müller cells. Although these data could provide an interesting insight into retinal gliosis mechanisms, further studies are needed to unravel the precise cellular and molecular mechanisms linking plasmalogen levels to GFAP and Cx43 protein metabolism. Several mechanisms can be hypothesized. First, GFAP and Cx43 have several common transcription factors regulating sites in their promoter sequence such as NFkB, AP-1 or AP-2 (Geimonen et al., 1996; Gomes et al., 1999; Wu et al., 2013; Brenner et al., 2019). Quantifying these transcriptional regulators could therefore be of great interest. Secondly, it has been shown that plasmalogens regulate Protein Kinase C (PKC) activity (Daniel et al., 1988; Sejimo et al., 2018), which is responsible for Cx43 phosphorylation and subsequent degradation by the ubiquitin-proteasome pathway (Leithe, 2016). Interestingly, GFAP is also regulated by several mechanisms including phosphorylation (Harrison and Mobley, 1991; Inagaki et al., 1994), and it have been suggested that phosphorylation status of GFAP in glial cells may play an important role in astrocyte remodeling during development and disease (Sullivan et al., 2012). Accordingly, we can hypothesize that plasmalogen depletion could induce a greater activity of PKC and then contribute at least partially to Cx43 and/or GFAP downregulation.

It can be underlined that our model of partial reduction of cellular plasmalogens presented in this study could be of particular interest to investigate the plasmalogen-related cellular mechanisms, as it may mimic biochemical alterations that are closer to pathological conditions when compared to fully-deficient cell lines, which are only relevant for rare diseases such as Rhizomelic chondrodysplasia punctata (RCDP).

To conclude, we show that cell levels of plasmalogens influence the expression and phosphorylation of Cx43, the major protein in gap junctions. In addition to the Cx43 downregulation, plasmalogen-depleted Müller cells displayed defects in both the ATP-stimulated calcium response (initiation) as well as calcium wave’s propagation to neighboring astrocytes. Finally, we show that plasmalogen depletion of Müller cells was associated with an alteration of their migration abilities, as well as a downregulation of GFAP expression, a glia-specific cytoskeleton protein involved in glial activation, but not with other cytoskeleton-related proteins. Therefore, our data support the hypothesis that plasmalogen metabolism is involved in the regulation of Cx43 in the retina and Müller cells, and regulates crucial functions of Müller cells such as GJIC and cell migration.

## Data Availability

The raw data supporting the conclusion of this article will be made available by the authors, without undue reservation.

## References

[B1] Acar N. Gregoire S. Andre A. Juaneda P. Joffre C. Bron A. M. (2007). Plasmalogens in the Retina: *In Situ* Hybridization of Dihydroxyacetone Phosphate Acyltransferase (DHAP-AT) - the First Enzyme Involved in Their Biosynthesis - and Comparative Study of Retinal and Retinal Pigment Epithelial Lipid Composition. Exp. Eye Res. 84 (1), 143–151. 10.1016/j.exer.2006.09.009 17081518

[B2] Begum G. Kintner D. Liu Y. Cramer S. W. Sun D. (2012). DHA Inhibits ER Ca2+ Release and ER Stress in Astrocytes Following *In Vitro* Ischemia. J. Neurochem. 120 (4), 622–630. 10.1111/j.1471-4159.2011.07606.x 22129278 PMC3259263

[B3] Brenner M. Messing A. Olsen M. L. (2019). AP‐1 and the Injury Response of the GFAP Gene. J. Neuro Res. 97 (2), 149–161. 10.1002/jnr.24338 PMC6289842 30345544

[B4] Bringmann A. Pannicke T. Grosche J. Francke M. Wiedemann P. Skatchkov S. (2006). Müller Cells in the Healthy and Diseased Retina. Prog. Retin. Eye Res. 25 (4), 397–424. 10.1016/j.preteyeres.2006.05.003 16839797

[B5] Butkevich E. Hülsmann S. Wenzel D. Shirao T. Duden R. Majoul I. (2004). Drebrin Is a Novel Connexin-43 Binding Partner that Links gap Junctions to the Submembrane Cytoskeleton. Curr. Biol. 14 (8), 650–658. 10.1016/j.cub.2004.03.063 15084279

[B6] Cabot M. C. Jaken S. (1984). Structural and Chemical Specificity of Diradylglycerols for Protein Kinase C Activation. Biochem. Biophysical Res. Commun. 125 (1), 163–169. 10.1016/s0006-291x(84)80349-6 6239621

[B7] Clark K. J. Murray A. W. (1995). Evidence that the Bradykinin-Induced Activation of Phospholipase D and of the Mitogen-Activated Protein Kinase cascade Involve Different Protein Kinase C Isoforms. J. Biol. Chem. 270 (13), 7097–7103. 10.1074/jbc.270.13.7097 7535766

[B8] Crespin S. Bechberger J. Mesnil M. Naus C. C. Sin W.-C. (2010). The Carboxy-Terminal Tail of Connexin43 gap junction Protein Is Sufficient to Mediate Cytoskeleton Changes in Human Glioma Cells. J. Cel. Biochem. 110 (3), 589–597. 10.1002/jcb.22554 20512920

[B9] Daniel L. W. Small G. W. Schmitt J. D. Marasco C. J. Ishaq K. Piantadosi C. (1988). Alkyl-linked Diglycerides Inhibit Protein Kinase C Activation by Diacylglycerols. Biochem. Biophysical Res. Commun. 151 (1), 291–297. 10.1016/0006-291x(88)90592-x 3348778

[B10] Dorman R. V. Dreyfus H. Freysz L. Horrocks L. A. (1976). Ether Lipid Content of Phosphoglycerides from the Retina and Brain of Chicken and Calf. Biochim. Biophys. Acta 486 (1), 55–59. 10.1016/0005-2760(77)90069-8 1009135

[B11] Folch J. Lees M. Stanley G. H. S. (1957). A Simple Method for the Isolation and Purification of Total Lipides from Animal Tissues. J. Biol. Chem. 226 (1), 497–509. 10.1016/s0021-9258(18)64849-5 13428781

[B12] Ganong B. R. Loomis C. R. Hannun Y. A. Bell R. M. (1986). Specificity and Mechanism of Protein Kinase C Activation by Sn-1,2-Diacylglycerols. Proc. Natl. Acad. Sci. U.S.A. 83 (5), 1184–1188. 10.1073/pnas.83.5.1184 3456578 PMC323039

[B13] Geimonen E. Jiang W. Ali M. Fishman G. I. Garfield R. E. Andersen J. (1996). Activation of Protein Kinase C in Human Uterine Smooth Muscle Induces Connexin-43 Gene Transcription through an AP-1 Site in the Promoter Sequence. J. Biol. Chem. 271 (39), 23667–23674. 10.1074/jbc.271.39.23667 8798588

[B14] Gomes F. C. A. Paulin D. Moura Neto V. (1999). Glial Fibrillary Acidic Protein (GFAP): Modulation by Growth Factors and its Implication in Astrocyte Differentiation. Braz. J. Med. Biol. Res. 32 (5), 619–631. 10.1590/s0100-879x1999000500016 10412574

[B15] Goodenough D. A. Goliger J. A. Paul D. L. (1996). Connexins, Connexons, and Intercellular Communication. Annu. Rev. Biochem. 65, 475–502. 10.1146/annurev.bi.65.070196.002355 8811187

[B16] Gould C. Newton A. (2008). The Life and Death of Protein Kinase C. Cdt 9 (8), 614–625. 10.2174/138945008785132411 PMC5783564 18691009

[B17] Guerrero-Valero M. Ferrer-Orta C. Querol-Audí J. Marin-Vicente C. Fita I. Gómez-Fernández J. C. (2009). Structural and Mechanistic Insights into the Association of PKCα-C2 Domain to PtdIns(4,5)P 2. Proc. Natl. Acad. Sci. U.S.A. 106 (16), 6603–6607. 10.1073/pnas.0813099106 19346474 PMC2672498

[B18] Harrison B. C. Mobley P. L. (1991). Phorbol Myristate Acetate and 8-Bromo-Cyclic AMP-Induced Phosphorylation of Glial Fibrillary Acidic Protein and Vimentin in Astrocytes: Comparison of Phosphorylation Sites. J. Neurochem. 56 (5), 1723–1730. 10.1111/j.1471-4159.1991.tb02073.x 2013762

[B19] Hervé J.-C. Derangeon M. (2013). Gap-junction-mediated Cell-To-Cell Communication. Cell Tissue Res. 352 (1), 21–31. 10.1007/s00441-012-1485-6 22940728

[B20] Heymans F. Da Silva C. Marrec N. Godfroid J.-J. Castagna M. (1987). Alkyl Analogs of Diacylglycerol as Activators of Protein Kinase C. FEBS Lett. 218 (1), 35–40. 10.1016/0014-5793(87)81013-x 3595862

[B21] Hicks D. Courtois Y. (1990). The Growth and Behaviour of Rat Retinal Müller Cells *In Vitro* 1. An Improved Method for Isolation and Culture. Exp. Eye Res. 51 (2), 119–129. 10.1016/0014-4835(90)90063-z 2387332

[B22] Homkajorn B. Sims N. R. Muyderman H. (2010). Connexin 43 Regulates Astrocytic Migration and Proliferation in Response to Injury. Neurosci. Lett. 486 (3), 197–201. 10.1016/j.neulet.2010.09.051 20869426

[B23] Inagaki M. IMakamura Y. Takeda M. Nishimura T. Inagaki N. (1994). Glial Fibrillary Acidic Protein: Dynamic Property and Regulation by Phosphorylation. Brain Pathol. 4 (3), 239–243. 10.1111/j.1750-3639.1994.tb00839.x 7952265

[B24] Kerr N. M. Johnson C. S. de Souza C. F. Chee K.-S. Good W. R. Green C. R. (2010). Immunolocalization of gap junction Protein Connexin43 (GJA1) in the Human Retina and Optic Nerve. Invest. Ophthalmol. Vis. Sci. 51 (8), 4028–4034. 10.1167/iovs.09-4847 20375327

[B25] Kimura K. Nishida T. (2010). Role of the Ubiquitin-Proteasome Pathway in Downregulation of the Gap-Junction Protein Connexin43 by TNF-α in Human Corneal Fibroblasts. Invest. Ophthalmol. Vis. Sci. 51 (4), 1943–1947. 10.1167/iovs.09-3573 19907029

[B26] Kurth-Nelson Z. L. Mishra A. Newman E. A. (2009). Spontaneous Glial Calcium Waves in the Retina Develop over Early Adulthood. J. Neurosci. 29 (36), 11339–11346. 10.1523/JNEUROSCI.2493-09.2009 19741140 PMC3148946

[B27] Lagos-Cabré R. Burgos-Bravo F. Avalos A. M. Leyton L. (2019). Connexins in Astrocyte Migration. Front. Pharmacol. 10, 1546. 10.3389/fphar.2019.01546 32009957 PMC6974553

[B28] Laird D. W. (2006). Life Cycle of Connexins in Health and Disease. Biochem. J. 394 (Pt 3), 527–543. 10.1042/BJ20051922 16492141 PMC1383703

[B29] Leithe E. Mesnil M. Aasen T. (2018). The Connexin 43 C-Terminus: A Tail of many Tales. Biochim. Biophys. Acta (Bba) - Biomembr. 1860 (1), 48–64. 10.1016/j.bbamem.2017.05.008 28526583

[B30] Leithe E. (2016). Regulation of Connexins by the Ubiquitin System: Implications for Intercellular Communication and Cancer. Biochim. Biophys. Acta (Bba) - Rev. Cancer 1865 (2), 133–146. 10.1016/j.bbcan.2016.02.001 26855059

[B31] Leithe E. Rivedal E. (2004). Ubiquitination and Down-Regulation of gap junction Protein Connexin-43 in Response to 12-O-Tetradecanoylphorbol 13-acetate Treatment. J. Biol. Chem. 279 (48), 50089–50096. 10.1074/jbc.M402006200 15371442

[B32] Liu D. Nagan N. Just W. W. Rodemer C. Thai T.-P. Zoeller R. A. (2005). Role of Dihydroxyacetonephosphate Acyltransferase in the Biosynthesis of Plasmalogens and Nonether Glycerolipids. J. Lipid Res. 46 (4), 727–735. 10.1194/jlr.m400364-jlr200 15687349

[B33] Lučić I. Truebestein L. Leonard T. A. (2016). Novel Features of DAG-Activated PKC Isozymes Reveal a Conserved 3-D Architecture. J. Mol. Biol. 428 (1), 121–141. 10.1016/j.jmb.2015.11.001 26582574

[B34] Mandal A. Wang Y. Ernsberger P. Kester M. (1997). Interleukin-1-induced Ether-Linked Diglycerides Inhibit Calcium-Insensitive Protein Kinase C Isotypes. J. Biol. Chem. 272 (32), 20306–20311. 10.1074/jbc.272.32.20306 9242712

[B35] Matsuuchi L. Naus C. C. (2013). Gap junction Proteins on the Move: Connexins, the Cytoskeleton and Migration. Biochim. Biophys. Acta (Bba) - Biomembr. 1828 (1), 94–108. 10.1016/j.bbamem.2012.05.014 22613178

[B36] Metea M. R. Newman E. A. (2006). Glial Cells Dilate and Constrict Blood Vessels: a Mechanism of Neurovascular Coupling. J. Neurosci. 26 (11), 2862–2870. 10.1523/JNEUROSCI.4048-05.2006 16540563 PMC2270788

[B37] Morrison W. R. Smith L. M. (1964). Preparation of Fatty Acid Methyl Esters and Dimethylacetals from Lipids with boron Fluoride-Methanol. J. Lipid Res. 5, 600–608. 10.1016/s0022-2275(20)40190-7 14221106

[B38] Nagan N. Zoeller R. A. (2001). Plasmalogens: Biosynthesis and Functions. Prog. Lipid Res. 40 (3), 199–229. 10.1016/s0163-7827(01)00003-0 11275267

[B39] Nagy K. Brahmbhatt V. V. Berdeaux O. Bretillon L. Destaillats F. Acar N. (2012). Comparative Study of Serine-Plasmalogens in Human Retina and Optic Nerve: Identification of Atypical Species with Odd Carbon Chains. J. Lipid Res. 53 (4), 776–783. 10.1194/jlr.D022962 22266369 PMC3307654

[B40] Newman E. A. (2015). Glial Cell Regulation of Neuronal Activity and Blood Flow in the Retina by Release of Gliotransmitters. Phil. Trans. R. Soc. B 370 (1672), 20140195. 10.1098/rstb.2014.0195 26009774 PMC4455764

[B41] Newman E. A. (2001). Propagation of Intercellular Calcium Waves in Retinal Astrocytes and Müller Cells. J. Neurosci. 21 (7), 2215–2223. 10.1523/jneurosci.21-07-02215.2001 11264297 PMC2409971

[B42] Newman E. A. Zahs K. R. (1997). Calcium Waves in Retinal Glial Cells. Science 275 (5301), 844–847. 10.1126/science.275.5301.844 9012354 PMC2410141

[B43] O′Sullivan M. L. Puñal V. M. Kerstein P. C. Brzezinski J. A. Glaser T. Wright K. M. (2017). Astrocytes Follow Ganglion Cell Axons to Establish an Angiogenic Template during Retinal Development. Glia 65 (10), 1697–1716. 10.1002/glia.23189 28722174 PMC5561467

[B44] Olk S. Turchinovich A. Grzendowski M. Stühler K. Meyer H. E. Zoidl G. (2009). Proteomic Analysis of Astroglial Connexin43 Silencing Uncovers a Cytoskeletal Platform Involved in Process Formation and Migration. Glia 58 (4), NA. 10.1002/glia.20942 19795503

[B45] Oyamada M. Takebe K. Oyamada Y. (2013). Regulation of Connexin Expression by Transcription Factors and Epigenetic Mechanisms. Biochim. Biophys. Acta (Bba) - Biomembr. 1828 (1), 118–133. 10.1016/j.bbamem.2011.12.031 22244842

[B46] Reichenbach A. Bringmann A. (2013). New Functions of Müller Cells. Glia 61 (5), 651–678. 10.1002/glia.22477 23440929

[B47] Rodemer C. Thai T.-P. Brugger B. Kaercher T. Werner H. Nave K.-A. (2003). Inactivation of Ether Lipid Biosynthesis Causes Male Infertility, Defects in Eye Development and Optic Nerve Hypoplasia in Mice. Hum. Mol. Genet. 12 (15), 1881–1895. 10.1093/hmg/ddg191 12874108

[B48] Saab S. Buteau B. Leclère L. Bron A. M. Creuzot-Garcher C. P. Bretillon L. (2014). Involvement of Plasmalogens in post-natal Retinal Vascular Development. PLoS One 9 (6), e101076. 10.1371/journal.pone.0101076 24963632 PMC4071069

[B49] Scemes E. Dermietzel R. Spray D. C. (1998). Calcium Waves between Astrocytes from Cx43 Knockout Mice. Glia 24 (1), 65–73. 10.1002/(sici)1098-1136(199809)24:1<65::aid-glia7>3.0.co;2-# 9700490 PMC1808224

[B50] Schildge S. Bohrer C. Beck K. Schachtrup C. (2013). Isolation and Culture of Mouse Cortical Astrocytes. JoVE 71, 50079. 10.3791/50079 PMC3582677 23380713

[B51] Sejimo S. Hossain M. S. Akashi K. (2018). Scallop-derived Plasmalogens Attenuate the Activation of PKCδ Associated with the Brain Inflammation. Biochem. Biophysical Res. Commun. 503 (2), 837–842. 10.1016/j.bbrc.2018.06.084 29920240

[B52] Sergeeva M. Strokin M. Reiser G. (2005). Regulation of Intracellular Calcium Levels by Polyunsaturated Fatty Acids, Arachidonic Acid and Docosahexaenoic Acid, in Astrocytes: Possible Involvement of Phospholipase A2. Reprod. Nutr. Dev. 45 (5), 633–646. 10.1051/rnd:2005050 16188212

[B53] Sullivan S. M. Sullivan R. K. P. Miller S. M. Ireland Z. Björkman S. T. Pow D. V. (2012). Phosphorylation of GFAP Is Associated with Injury in the Neonatal Pig Hypoxic-Ischemic Brain. Neurochem. Res. 37 (11), 2364–2378. 10.1007/s11064-012-0774-5 22528834

[B54] Tchernookova B. K. Heer C. Young M. Swygart D. Kaufman R. Gongwer M. (2018). Activation of Retinal Glial (Müller) Cells by Extracellular ATP Induces Pronounced Increases in Extracellular H+ Flux. PLoS One 13 (2), e0190893. 10.1371/journal.pone.0190893 29466379 PMC5821311

[B55] Todt H. Dorninger F. Rothauer P. J. Fischer C. M. Schranz M. Bruegger B. (2020). Oral Batyl Alcohol Supplementation Rescues Decreased Cardiac Conduction in Ether Phospholipid‐deficient Mice. Jrnl Inher Metab. Disea 43 (5), 1046–1055. 10.1002/jimd.12264 PMC7540404 32441337

[B56] Toft-Kehler A. K. Skytt D. M. Kolko M. (2018). A Perspective on the Müller Cell-Neuron Metabolic Partnership in the Inner Retina. Mol. Neurobiol. 55 (6), 5353–5361. 10.1007/s12035-017-0760-7 28929338

[B57] Uckermann O. Grosche J. Reichenbach A. Bringmann A. (2002). ATP-evoked Calcium Responses of Radial Glial (Müller) Cells in the Postnatal Rabbit Retina. J. Neurosci. Res. 70 (2), 209–218. 10.1002/jnr.10406 12271470

[B58] Vecino E. Rodriguez F. D. Ruzafa N. Pereiro X. Sharma S. C. (2016). Glia-neuron Interactions in the Mammalian Retina. Prog. Retin. Eye Res. 51, 1–40. 10.1016/j.preteyeres.2015.06.003 26113209

[B59] Vine A. L. Leung Y. M. Bertram J. S. (2005). Transcriptional Regulation of Connexin 43 Expression by Retinoids and Carotenoids: Similarities and Differences. Mol. Carcinog. 43 (2), 75–85. 10.1002/mc.20080 15754312

[B60] Warne T. R. Buchanan F. G. Robinson M. (1995). Growth-dependent Accumulation of Monoalkylglycerol in Madin-Darby Canine Kidney Cells. J. Biol. Chem. 270 (19), 11147–11154. 10.1074/jbc.270.19.11147 7744745

[B61] Wu X. Huang W. Luo G. Alain L. A. (2013). Hypoxia Induces Connexin 43 Dysregulation by Modulating Matrix Metalloproteinases via MAPK Signaling. Mol. Cel Biochem. 384 (1-2), 155–162. 10.1007/s11010-013-1793-5 PMC3825321 24002703

[B62] Zahs K. R. Ceelen P. W. (2006). Gap Junctional Coupling and Connexin Immunoreactivity in Rabbit Retinal Glia. Vis. Neurosci. 23 (1), 1–10. 10.1017/S0952523806231018 16597346

