# Supplementary material for: Plasmalogens Regulate Retinal Connexin 43 Expression and Müller Glial Cells Gap Junction Intercellular Communication and Migration
Source: Front Cell Dev Biol. 2022 Mar 31;10:864599. doi: 10.3389/fcell.2022.864599 (PMC9009447; doi:10.3389/fcell.2022.864599)
Supplement: Supplementary file 1 [file Presentation1.PPTX]

## Slide 1
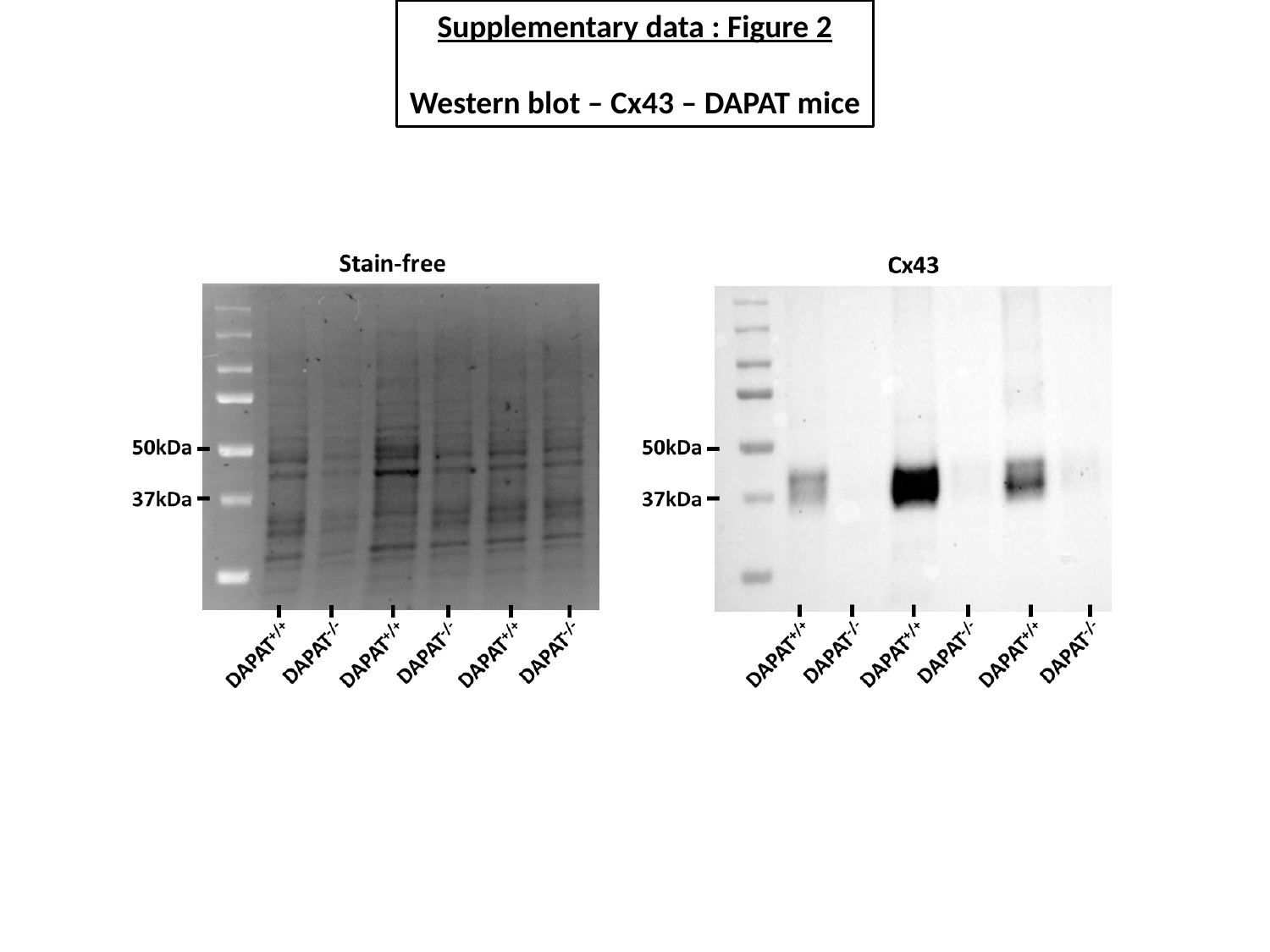

Supplementary data : Figure 2
Western blot – Cx43 – DAPAT mice

## Slide 2
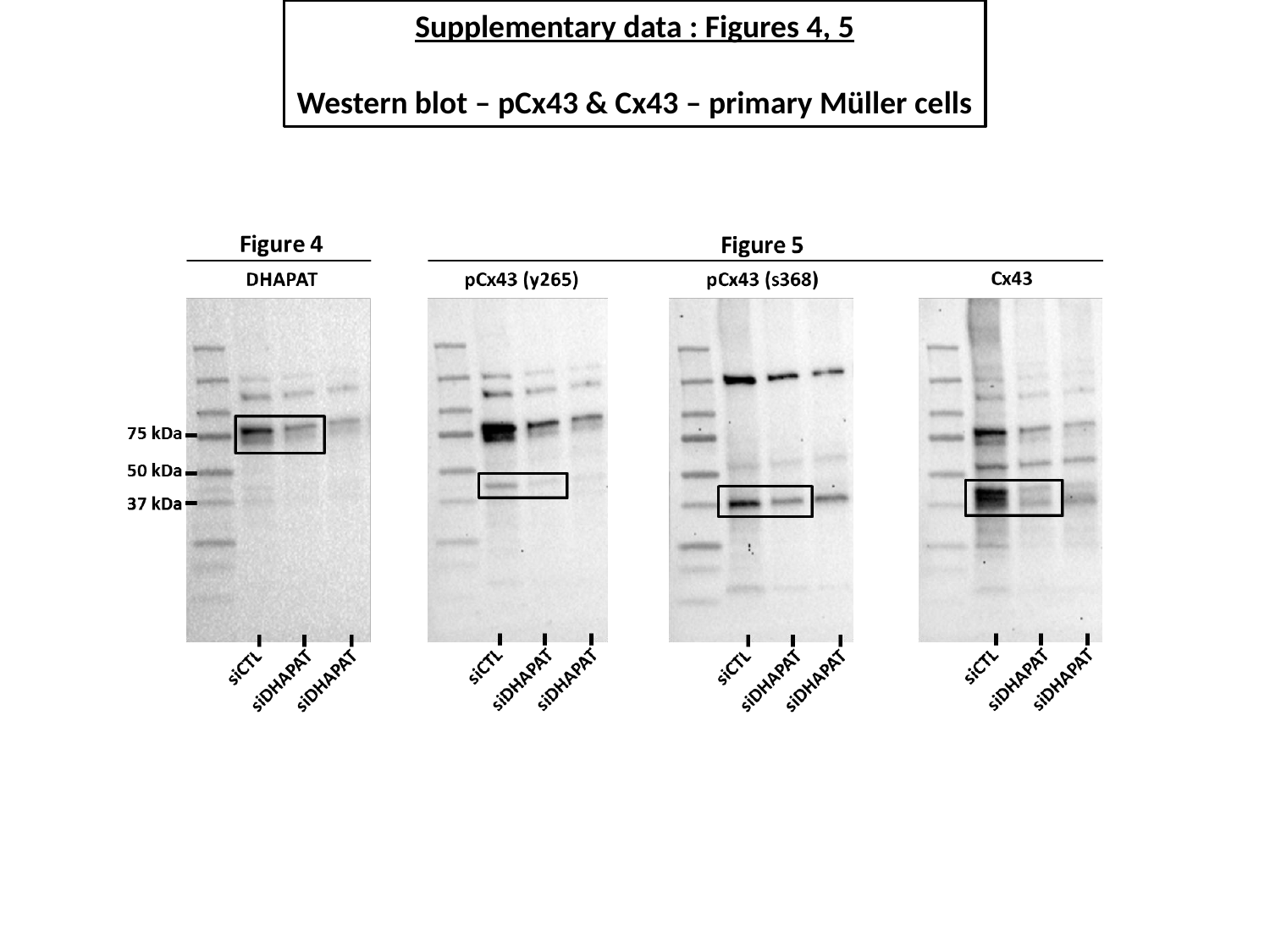

Supplementary data : Figures 4, 5
Western blot – pCx43 & Cx43 – primary Müller cells

## Slide 3
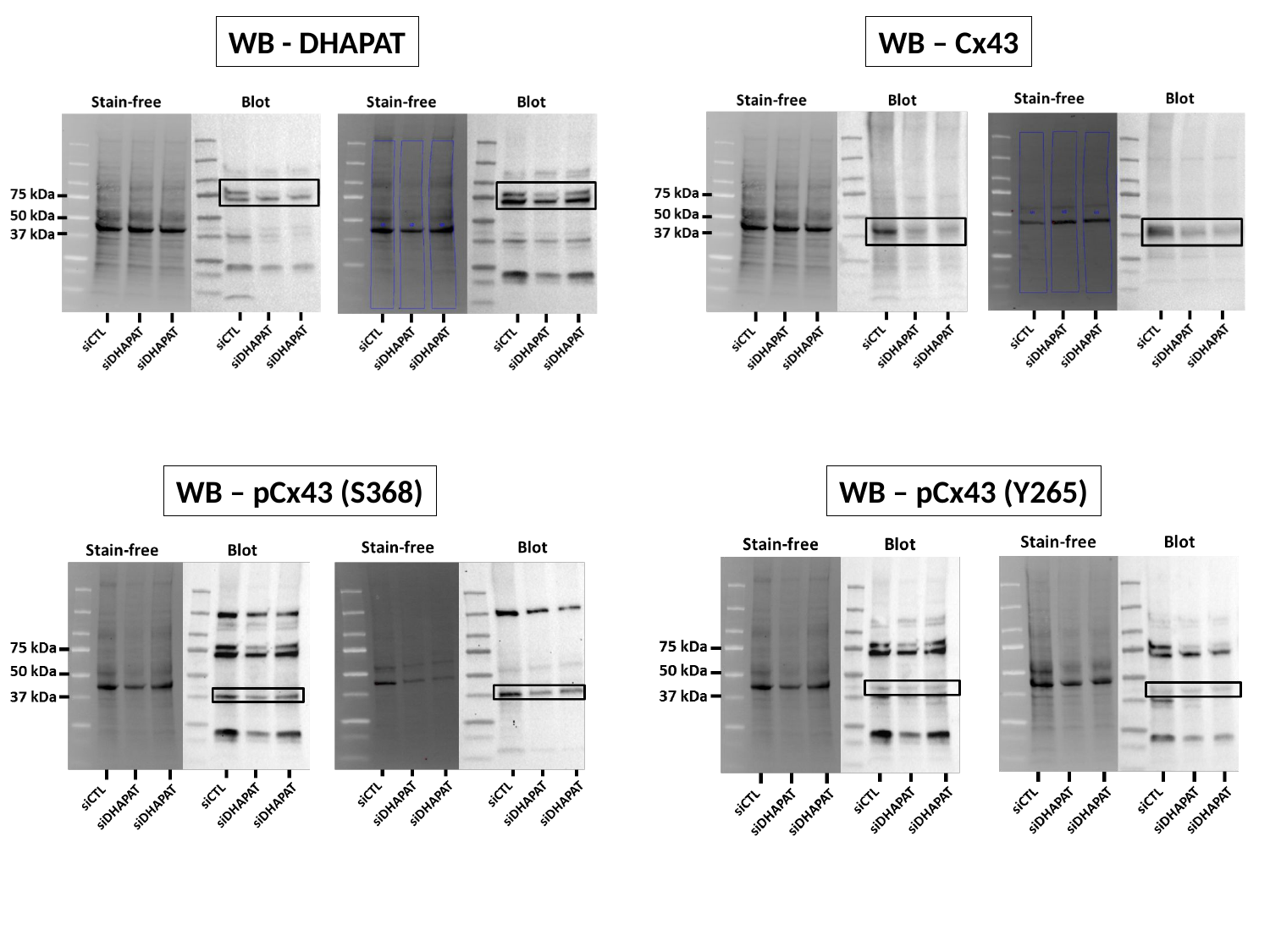

WB - DHAPAT
WB – Cx43
WB – pCx43 (S368)
WB – pCx43 (Y265)

## Slide 4
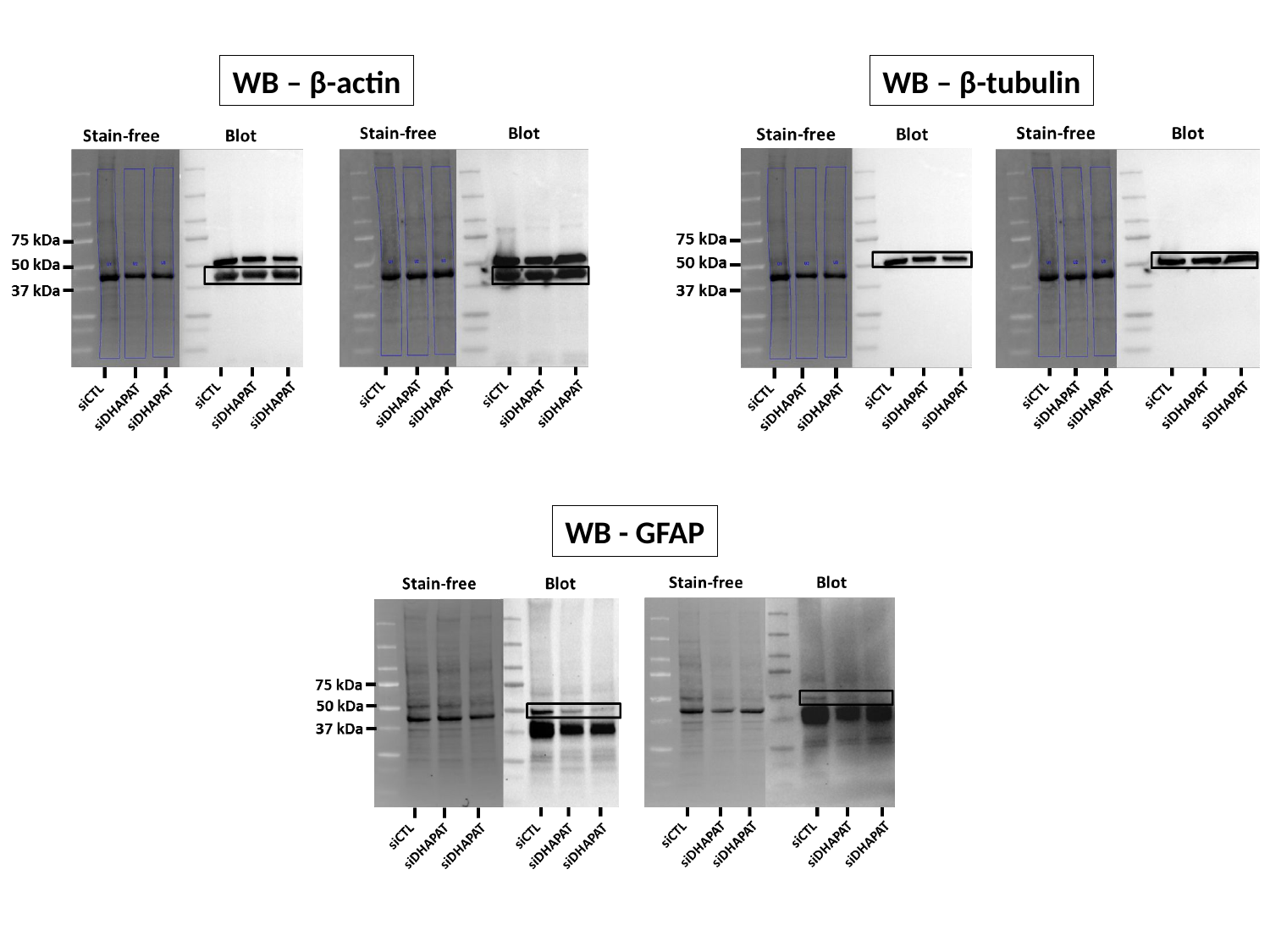

WB – β-actin
WB – β-tubulin
WB - GFAP
